# Non-linear, *cata*-Condensed, Polycyclic Aromatic Hydrocarbon Materials: A Generic Approach and Physical Properties

**DOI:** 10.1002/chem.201501861

**Published:** 2015-06-08

**Authors:** Barnaby T Haire, Kane W J Heard, Mark S Little, Adam V S Parry, James Raftery, Peter Quayle, Stephen G Yeates

**Affiliations:** [a] School of Chemistry, University of ManchesterManchester M13 9PL (UK)

**Keywords:** benzannulation, coupling, cyclization, polycyclic aromatic hydrocarbons, polycycles

## Abstract

A generic approach to the regiospecific synthesis of halogenated polycyclic aromatics is made possible by the one- or two-directional benzannulation reactions of readily available (*ortho*-allylaryl)trichloroacetates (the “BHQ” reaction). Palladium-catalysed cross-coupling reactions of the so-formed haloaromatics enable the synthesis of functionalised polycyclic aromatic hydrocarbons (PAHs) with surgical precision. Overall, this new methodology enables the facile mining of chemical space in search of new electronic functional materials.

To date, linearly annulated acenes such as tetracene and pentacene **1** (Figure 1) represent the most-studied class of carbon-based small-molecule electronic materials.[1a,b] Representative members of this class of PAHs include rubrene[2] and TIPS-pentacene (TIPS=triisopropylsilyl),[3] both of which possess excellent electronic and morphological properties for the construction of organic field-effect transistor (OFET) devices. In recent years “non-acene”, *peri*-condensed PAHs which incorporate either a perylene, **2**, or pyrene[4a,b] structural motif have also been the subject of increasing interest, a situation which lies in contrast to non-linear *cata*-PAHs whose potential as electronic materials is comparatively unexplored. Recently, it has been shown that the larger phenacenes possess high mobility hole transport behaviour in OFET devices.[5] Clar’s empirical rule of sextets dictates that acenes and phenacenes occupy two extremes of the PAH stability continuum.[6] Between these two isomeric forms lies a middle ground where HOMO–LUMO band gap and stability may be balanced while providing an extended π-system for efficient intermolecular charge transport. These prospects have resulted in increased interest in alternative PAH topologies in recent years.[7a,b]

**Figure 1:**
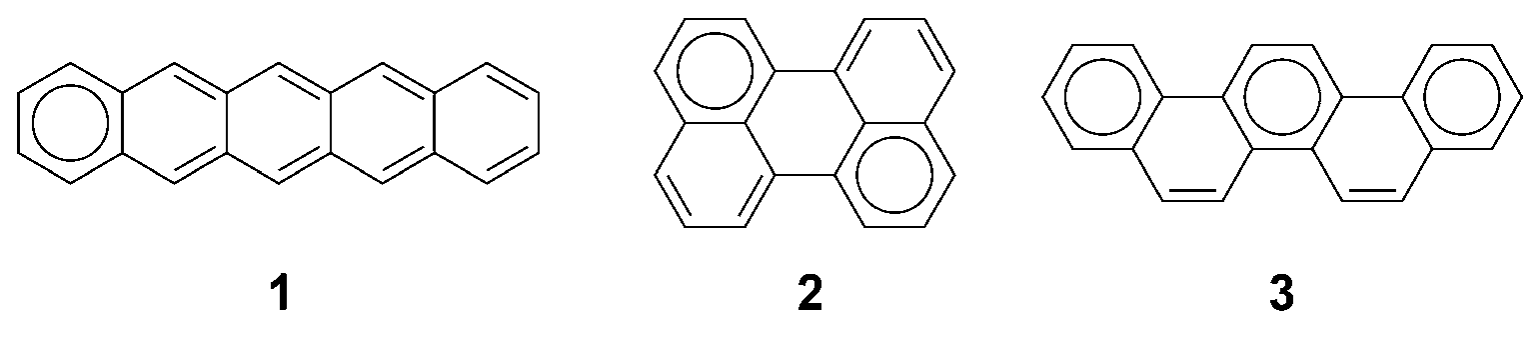
5-ring representatives of three classes of PAHs: pentacene 1 (acene), perylene 2 (*peri*-condensed) and picene 3 (phenacene).

In the light of this we recently embarked upon a programme of research which was tasked with the synthesis of *cata*-condensed PAHs and the evaluation of their electronic properties. As a starting point[8a] we elected to prepare 4,10-dichlorochrysene, **5**, starting from 1,5-dihydroxynaphthalene, **4**, using our newly discovered “BHQ” benzannulation reaction[9a] (Figure 2). In the event, **5** proved to be readily available by this route and served as a versatile scaffold, enabling the regiocontrolled synthesis of a small library of tetrasubstituted chrysene derivatives utilising S_N_Ar, Suzuki, Kumada, Sonogashira, Ullmann and Ir-catalysed C=H activation chemistries.

**Figure 2:**
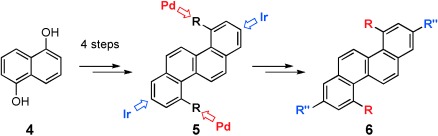
Synthesis of chrysene derivatives.

We now report that related benzannulation sequences provide a generic approach to a range of PAHs, extending the available chemical space that can be effectively explored. Etherification of 2,7-dihydroxynaphthalene **7** afforded the bis-allyl ether **8**, which underwent an *ortho*-Claisen rearrangement under unusually mild conditions (pyridine; 160 °C) to the bis-phenol **9** in 80 % yield. Trichloroacetylation of **9** to **10**, followed by mild thermolysis (diglyme at 162 °C) in the presence of CuCl (5 mol %) afforded the benzo[c]phenanthrene derivative **11** (Scheme 1) as a crystalline solid after filtration through a silica plug.

**Scheme 1:**
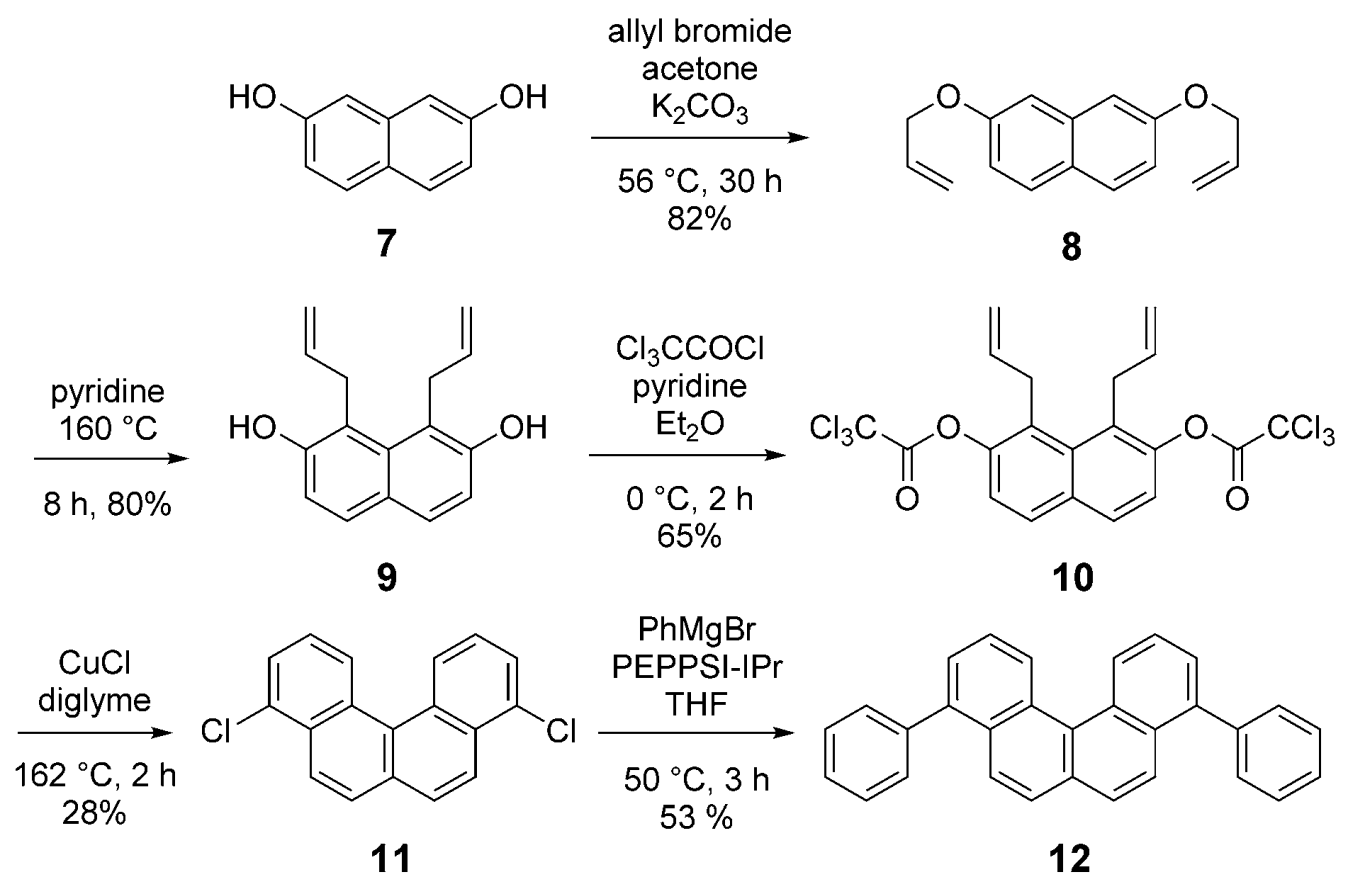
Synthesis of 4,9-benzo[c]phenanthrene derivatives.

The dichloride **11** underwent a representative palladium-catalysed cross-coupling with PhMgBr, affording **12** in 53 % yield. X-ray diffraction studies indicate that **11** adopts a columnar 1D π–π stacking structure. The molecule is twisted about the central naphthalene core in order to minimise steric interactions between the two fjord hydrogens. The shortest measureable C=C distance is 3.79 Å, which may be a result of Cl=Cl interactions, which are also 3.79 Å. The coupled product **12** also exhibits considerable distortion about the central core, whereas the introduction of the aryl residues at C-4/C-9 has little impact on the overall molecular geometry.

In the solid state **12** prefers to adopt a slip-stack arrangement where neighbouring molecules face in alternating directions, a packing regimen which results in short C=C contacts. DFT analysis (B3LYP/6-31+G(d)) of **11** indicates that the lone pairs on the chlorine substituents contribute significantly to the HOMO, whereas the dihedral angle of 56.6° about the bi-aryl axis in **12** allows some communication from the core to HOMO and LUMO of the peripheral aromatic substituents.

Having established that the BHQ benzannulation reaction provides rapid access to [4]catafusenes the synthesis of higher homologues was then pursued. To this end, alkylation of 2-bromo-α-tetralone **14** (Scheme 2) with 1-naphthol **15**, followed by Wittig olefination afforded the exocyclic alkene **16**. The *ortho*-Claisen rearrangement of **16** also proceeded under unusually mild conditions (pyridine; 115 °C; 2 h), and afforded **17** in essentially quantitative yield. Trichloroacetylation of **17** generated the key intermediate **18**, which, upon benzannulation (diglyme; CuCl, 5 mol %; 162 °C; 50 %), afforded **19**, presumably the result of an initial 8-*endo-trig-*cyclisation pathway (**TS A**). Dehydrogenation of **19** (DDQ, 2 equiv; *o*-DCB; 150 °C; 30 min) afforded isomerically pure **20**, a colourless, crystalline solid in 65 % yield. Palladium-catalysed cross-coupling of **20** with PhMgBr afforded **21** in 71 % yield, demonstrating the synthetic utility of these chlorinated PAHs.

**Scheme 2:**
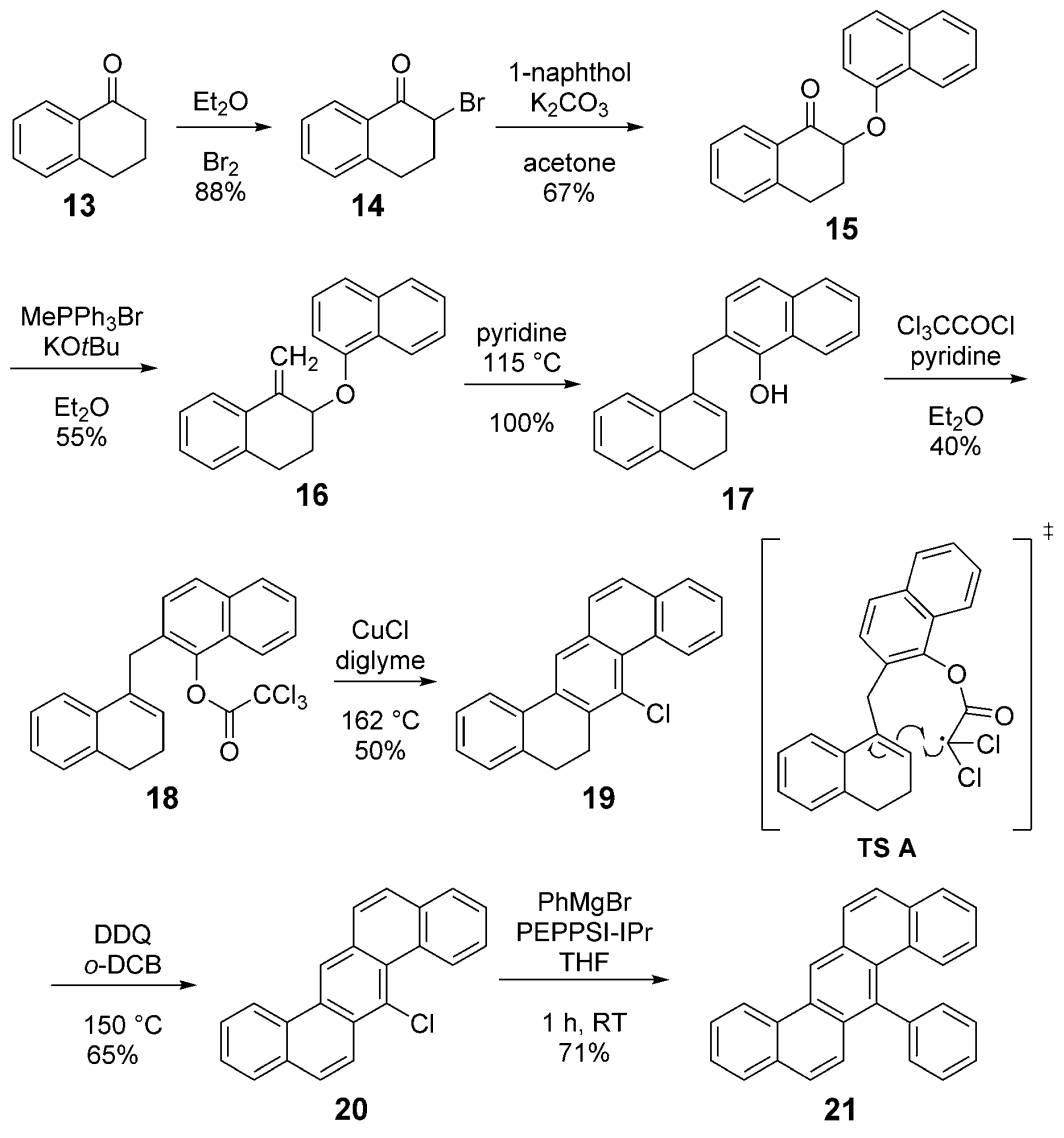
Synthesis of 7-benzo[k]tetraphene derivatives.

The XRD data and DFT MO plots for **20** and **21** are presented in Figure 3. Chloride **20** adopts a dimeric-herringbone packing arrangement, where pairs of molecules are held together cofacially at the van der Waals distance, stabilised by the asymmetric polarisation afforded by the chlorine substituent. Interdimeric interactions are largely CH-π in nature, resulting in a shortest C=C interatomic distance of 3.59 Å. Similarly, **21** packs as a dimeric aggregate in the solid state, and again exhibits an extensive network of CH-π interactions. The phenyl substituents in **21** are essentially orthogonal to the plane containing the central core, a structural feature which results in poor interaction between the respective sets of MOs, which is also evident in the solution-state electronic properties of this molecule.

**Figure 3:**
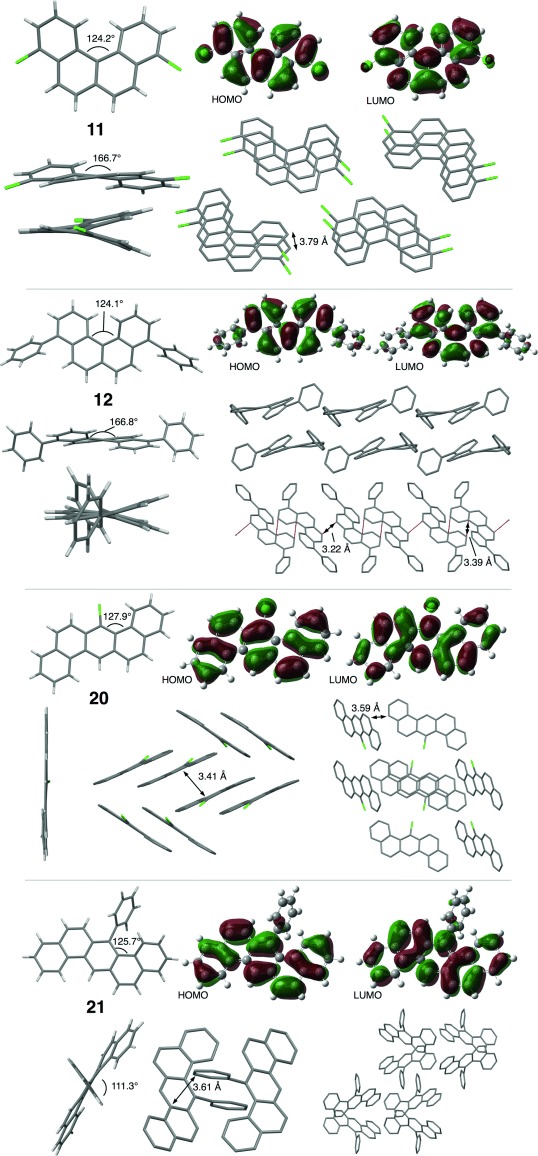
XRD structure, crystal packing and DFT MO plots for 11, 12, 20 and 21.

The potential of this new methodology was underscored by the development of a two-directional BHQ reaction in the synthesis of 7,17-dichlorodinaphtho[1,2,-b:1′,2′-k]chrysene, **27**, an example of a higher *cata*-PAH for which there is no general synthetic strategy.[6] Etherification of **14** with 1,5-dihydroxynaphthalene afforded diketone **22**, which, upon Wittig olefination to **23** and subsequent double *ortho*-Claisen rearrangement (pyridine; 115 °C; μW reactor), afforded the oxygen sensitive bis-phenol **24**. Immediate trichloroacetylation of **24** afforded the stable bis-trichloroacetate **25**, a high-melting crystalline solid in multi-gram quantities. The BHQ reaction of **25** leading to **26** required some optimisation, and was best effected using microwave activation in the prescence of the pre-formed copper-NHC complex **29** as catalyst[9b] (DCE; **29**, 5 mol %; 200 °C; μW reactor; 2 h; 87 %; see Scheme 3).

**Scheme 3:**
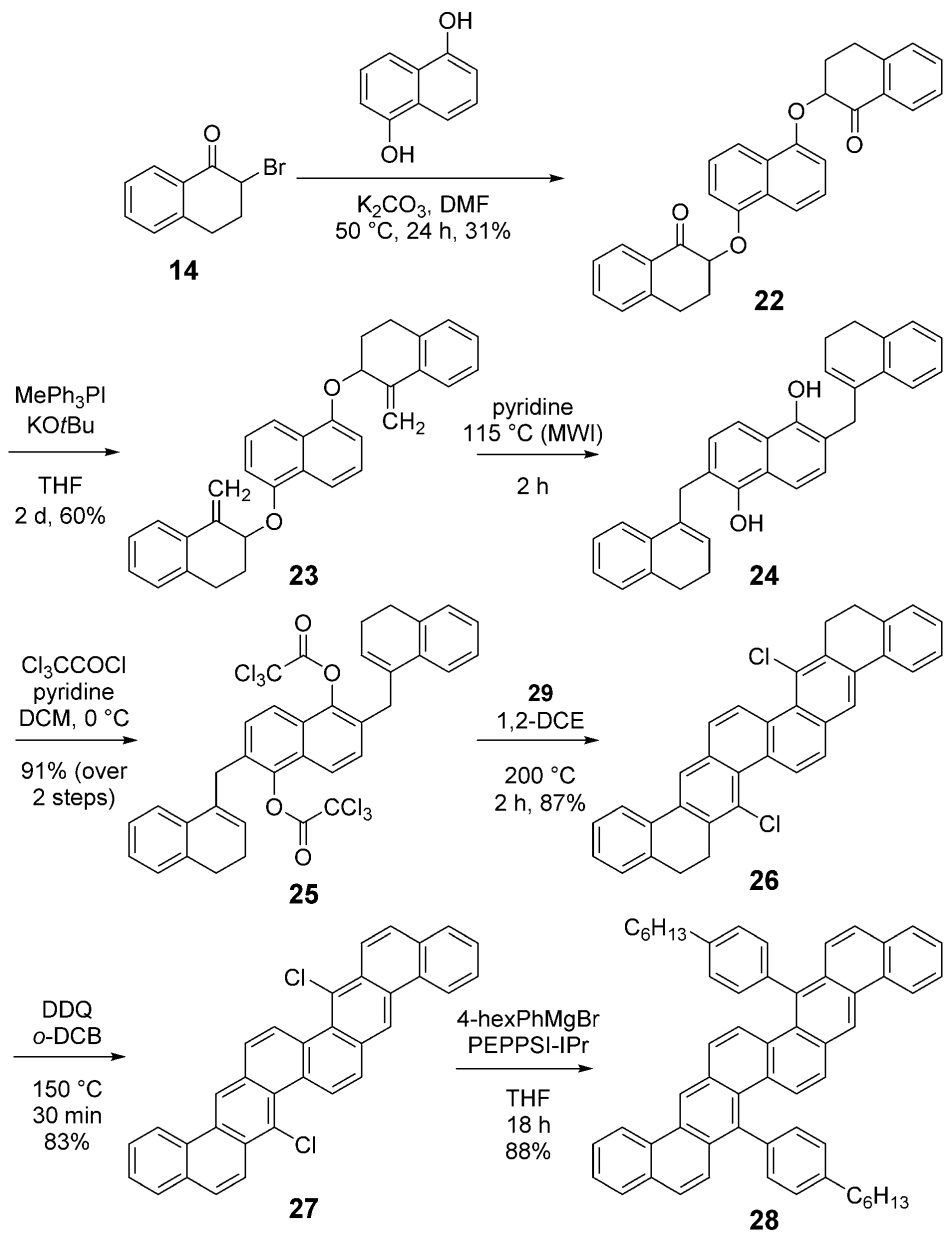
Synthesis of 7,17-dinaphtho[1,2,-b:1′,2′-k]chrysene derivatives. MWI=microwave irradiation.

Dehydrogenation of **26** (DDQ, 3 equiv; *o*-DCB; 150 °C; μW reactor; 30 min; 83 %) furnished the target PAH **27**, a compound which is insoluble at ambient temperatures. Remarkably, despite this lack of solubility, **27** underwent a Kumada–Corriu coupling with a simple solubilising group using Organ’s PEPPSI-IPr catalyst, affording **28** in excellent isolated yield. Gratifyingly **28** exhibited good solubility and solid-phase stability, characteristics which are essential for device fabrication. Recrystallisation of **27** from *o*-DCB at 181 °C eventually afforded material suitable for crystallographic analysis. This analysis revealed that **27** adopts a columnar π–π stacking morphology (Figure 4) with edge–face interactions between adjacent stacks, whereas DFT calculations indicated that the non-linear annulation mode in **27** lessens the degree of conjugation of the terminal rings with the central core.

**Figure 4:**
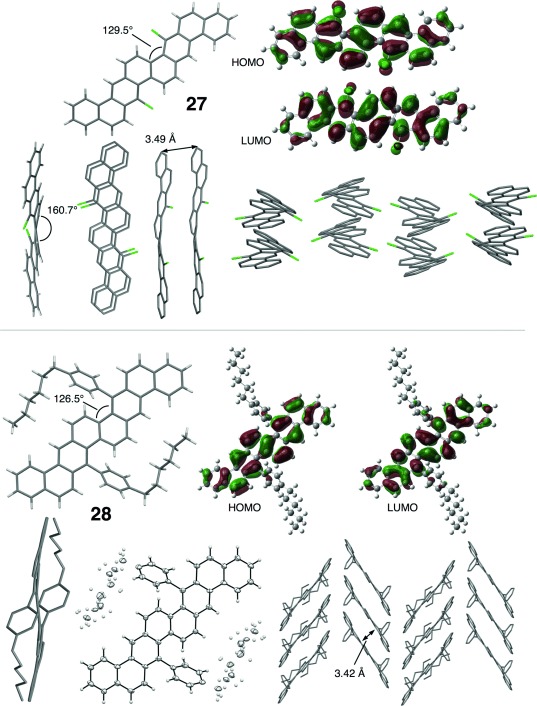
XRD structure, crystal packing and DFT MO plots for 27 and 28.

The XRD structure of **28** (Figure 4) reveals a symmetrical twist about the central bond (**7 b**–**17 b**), but the hexyl side-chains are disordered in one of the two molecules in the unit cell. The crystal structure is of a π-stacked herringbone type, with a contact spacing of 3.42 Å across a large proportion of the core. Predictably, the 4-hexylphenyl substituents preclude long-axis edge interaction between molecules. Biaryl and alkyl substituents typically disrupt charge-transfer pathways in at least one dimension.

The electronic data for cpds. **5**, **6**, **11**, **12**, **20**, **21**, **27** and **28** is tabulated below (Table 1). The energy of the HOMO–LUMO transition is typically estimated from the absorption edge; however, the large extinction disparity between the most intense absorptions and the lowest energy absorptions may cause inconsistencies in these measurements. It is to be noted that the highest energy fluorescence maximum *λ*_FluorMAX_ is a very close match for *λ*_EDGE_ and this could provide a less equivocal measure of the lowest energy optical transition. The absorption spectra of these compounds all share common features: a high extinction, short wavelength absorption, followed by a series of less intense long wavelength bands. The helicene-type morphology of **11** and **12** results in a slightly smaller optical gap than in phene-type chrysenes, but still much greater than that for tetracene (2.57 eV).[10] Likewise, extension of the chromophore to five and eight rings contracts the HOMO–LUMO much less than for the acenes, reflecting the increased benzenoid character of these systems.

**Table 1:** Absorption, fluorescence and voltammetry data for compounds 5, 6, 11, 12, 20, 21, 27 and 28.

Compound		*λ*_MAX_	*λ*_EDGE_		*λ*_FluorMAX_		*λ*_FluorMAX_-*λ*_EDGE_		Stokes		*V*_OX_		*E*_HOMO_^[b]^	*E*_LUMO_^[c]^
	No.	[nm]	[eV]	[nm]	[eV]	[nm]	[eV]	[nm]		[eV]	[nm]		wrt *V*_Fc_^[a]^	
Cl_2_-Chry	**5**	270	4.59	380	3.26	382	3.25	2	0.01	112	1.574	1.253	−6.05	−2.79
Ph_2_-Chry	**6**	288	4.31	376	3.30	385	3.22	9	0.08	97	1.439	1.042	−5.84	−2.54
Cl_2_-B[c]P	**11**	293	4.23	391	3.17	394	3.14	3	0.02	101	1.338	1.013	−5.81	−2.64
Ph_2_-B[c]P	**12**	297	4.17	393	3.15	394	3.14	1	0.01	97	1.453	1.062	−5.86	−2.71
Cl-B[k]T	**20**	303	4.09	410	3.02	408	3.03	−2	−0.01	105	1.479	1.076	−5.88	−2.86
Ph-B[k]T	**21**	302	4.11	409	3.03	406	3.05	−3	−0.02	104	1.197	0.930	−5.73	−2.70
Cl_2_-DNC	**27**	341	3.64	454	2.73	446	2.78	−8	−0.05	105	1.088^[d]^	0.800^[d]^	−5.60	−2.87
HexPh_2_-DNC	**28**	341	3.64	449	2.76	440	2.81	−9	−0.05	99	1.069	0.692	−5.49	−2.73

[a] Oxidation potentials measured from peak cathodic current relative to ferrocene. [b] *E*_HOMO_ estimated from *V*_OX_ where *V*_Fc_=−4.8 V. [c] *E*_LUMO_ estimated from *E*_HOMO_ and *λ*_EDGE_, [d] Oxidation potential for **27** measured from peak anodic current due to poor resolution of cathodic wave.

A comparison of the frontier MO energies of these compounds with representative organic semiconductor materials is depicted in Figure 5. The work function of the most common hole-injection electrode, gold, is also included as a reference point. Of note is the observation that **28** possesses a HOMO level approaching the range for efficient *p*-type behaviour, comparing favourably to high mobility material DNTT (Figure 6).

**Figure 5:**
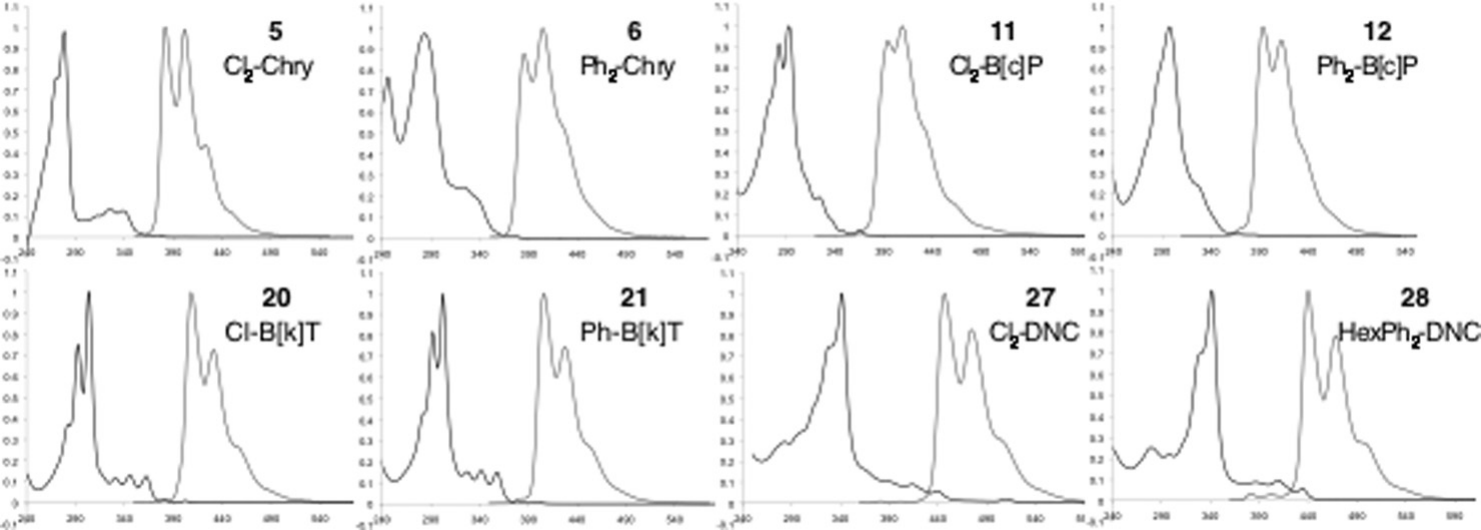
Normalised absorption (black) and fluorescence (grey) spectra for PAH derivatives. x: nm, y: arbitrary units.

**Figure 6:**
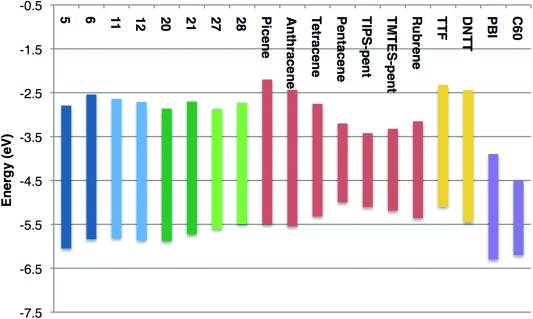
HOMO/LUMO energy levels of novel PAH derivatives compared with those of popular *p*-type PAH (red), sulfur-containing (gold) and *n*-type (violet) organic semiconductor materials (OSC) materials. Data from ref. [11] and references therein.

Field-effect transistors, fabricated by vacuum deposition of **28** onto an octadecyltrichlorosilane-treated SiO_2_ substrate, were characterised by XRD and AFM (Figure 7). These studies reveal the existence of small crystal domains of 0.25 μm^2^ with a roughness of 2.1 nm. The XRD indicates that the molecule is tilted toward the surface, normally at 36°. The devices showed good transistor behaviour, with an average saturation mobility of (0.03±0.01) cm^2^ V^−1^ s^−1^, a threshold of −9±1 V, on/off ratio of 4×10^6^ and a subthreshold swing of 600 mV dec^−1^. Further optimisation of the fabrication conditions to improve grain size and reduce the tilt angle could significantly improve device performance.

**Figure 7:**
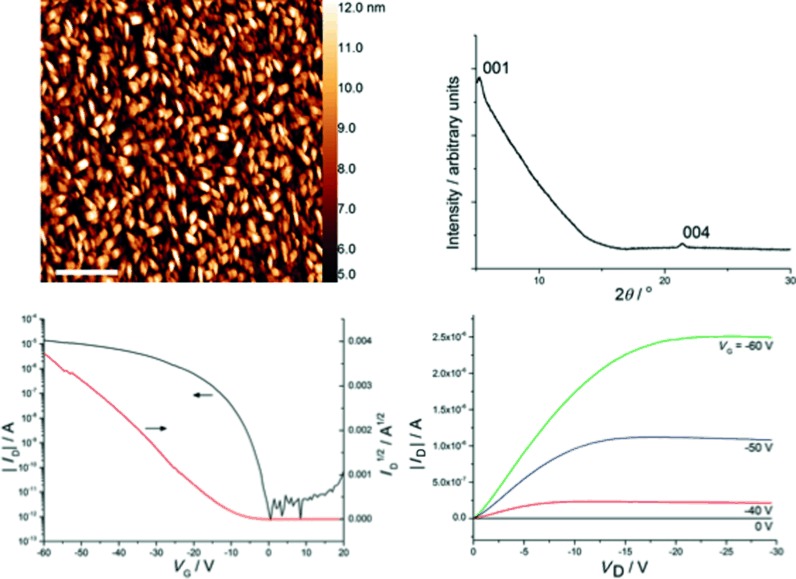
AFM and XRD characterisation of thin films of 28 with transfer and output curves for OFET devices.

In conclusion, we have developed a synthetic methodology that, for the first time, enables the regiocontrolled preparation of functionalised higher *cata*-fused PAHs with intermediate benzenoid character. This enables the mining of new chemical space facilitating the identification of PAHs possessing high intermolecular π–π overlap while maintaining chemical stability and solubility. Application of this methodology to the synthesis of new electronic materials is now underway.
