# Supplementary material for: Non-linear, cata-Condensed, Polycyclic Aromatic Hydrocarbon Materials: A Generic Approach and Physical Properties
Source: Chemistry. 2015 Jun 8;21(28):9970–4. doi: 10.1002/chem.201501861 (PMC4515081; doi:10.1002/chem.201501861)
Supplement: Supplementary file 1 [file chem0021-9970-sd1.pdf]

CHEMISTRY
A European Journal

Supporting Information

Non-linear, *cata*-Condensed, Polycyclic Aromatic Hydrocarbon Materials: A Generic Approach and Physical Properties

Barnaby T. Haire, Kane W. J. Heard, Mark S. Little,* Adam V. S. Parry, James Raftery, Peter Quayle,* and Stephen G. Yeates*[a]

chem_201501861_sm_miscellaneous_information.pdf

CARE: All polyclic aromatic hydrocarbons should be viewed as being toxic. The synthesis and manipulation of these compounds should be conducted in a well ventilated fume cupboard. Workers should always use appropriate safeguards so that these compounds do not come into contact with the skin. Avoid ingestion and inhalation. These compounds should be viewed as being toxic and having potential carcinogenic and mutagenic activities.

1 - General Experimental Procedure 4

2 - Synthetic Procedures

2.1 –Benzo[c]phenanthrenes

2,7-Bis(allyloxy)naphthalene (**7**) 5

1,8-Diallyl-2,7-dihydroxynaphthalene (**8**) 6

1,8-Diallyl-2,7-bis(2,2,2-trichloroacetyl)naphthalene (**9**) 7

4,9-Dichlorobenzo[c]phenanthrene (**10**) 8

4,9-Diphenylbenzo[c]phenanthrene (**11**) 9

2.2 –Benzo[k]tetraphenes

2-Bromo-1-tetralone (**14**) 10

2-(Naphthalene-1′-yloxy)-1-tetralone (**15**) 11

2′-(Naphthalene-1′-yloxy)-1-methylene-2,3,4-trihydronaphthalene (**16**) 12

2′-((3,4-Dihydronaphthalen-1-yl)methyl) naphthalen-1′-ol (**17**) 13

2′-((3,4-Dihydronaphthalen-1-yl)methyl) naphthalen-1′-trichloroacetate (**18**) 14

7-Chloro-5,6-dihydrobenzo[k]tetraphene (**19**) 15

7-Chlorobenzo[*k*]tetraphene (**20**) 16

7-Phenylbenzo[*k*]tetraphene (**21**) 17

2.3 – Dinaphtho[1,2,-b:1',2'-k]chrysenes

1,5-Bis(2-tetralonoxy)naphthalene (**22**) 18

1′,5′-Bis((1-methylene-1,2,3,4-tetrahydronaphthalen-2-yl)oxy) naphthalene (**23**) 19

2′,6′-Bis((3,4-dihydronaphthalen-1-yl)methyl) naphthalene-1′,5′-diol (**24**) 20

2′,6′-Bis((3,4-dihydronaphthalen-1-yl)methyl) naphthalene-1′,5′-bistrichloroacetate (**25**) 21

7,17-Dichloro-5,6,15,16-tetrahydrodinaphtho 22

[1,2,-*b*:1',2'-*k*]chrysene (**26**)
7,17-Dichlorodinaphtho[1,2,-*b*:1',2'-*k*]chrysene (**27**) 23
7,17-Bis(4-hexylphenyl)dinaphtho[1,2,-b:1',2'-k]chrysene (**28**) 24
3 - UV/vis Spectra
3.1 - 4,10-Dichlorochrysene (**5**) 25
3.2 – 4,10-Diphenylchrysene (**6**) 25
3.3 - 4,9-Dichlorobenzo[c]phenanthrene (**11**) 26
3.4 - 4,9-Diphenylbenzo[c]phenanthrene (**12**) 26
3.5 - 7-Chlorobenzo[k]tetraphene (**20**) 27
3.6 - 7-Phenylbenzo[k]tetraphene (**21**) 27
3.7 - 7,17-Dichlorodinaphtho[1,2,-b:1',2'-k]chrysene (**27**) 28
3.8 - 7,17-Bis(4-hexylphenyl)dinaphtho[1,2,-b:1',2'-k]chrysene (**28**) 28
4 - CV Data
4.1 - 4,10-Dichlorochrysene (**5**) 29
4.2 – 4,10-Diphenylchrysene (**6**) 29
4.3 - 4,9-Dichlorobenzo[c]phenanthrene (**11**) 30
4.4 - 4,9-Diphenylbenzo[c]phenanthrene (**12**) 30
4.5 - 7-Chlorobenzo[k]tetraphene (**20**) 31
4.6 - 7-Phenylbenzo[k]tetraphene (**21**) 31
4.7 - 7,17-Dichlorodinaphtho[1,2,-b:1',2'-k]chrysene (**27**) 32
4.8 - 7,17-Bis(4-hexylphenyl)dinaphtho[1,2,-b:1',2'-k]chrysene (**28**) 32
5 - NMR Spectra
4,10-Dichlorochrysene (**5**) 33
4,10-Diphenylchrysene (**6**) 34
2-Bromo-1-tetralone (**14**) 35
2-(Naphthalene-1′-yloxy)-1-tetralone (**15**) 36
2′-(Naphthalene-1′-yloxy)-1-methylene-2,3,4-trihydronaphthalene (**16**) 37
2′-((3,4-Dihydronaphthalen-1-yl)methyl) naphthalen-1′-ol (**17**) 38
2′-((3,4-Dihydronaphthalen-1-yl)methyl) naphthalen-1′-trichloroacetate (**18**) 39
7-Chloro-5,6-dihydrobenzo[k]tetraphene (**19**) 40
7-Chlorobenzo[k]tetraphene (**20**) 41
7-Phenylbenzo[k]tetraphene (**21**) 42

1,5-Bis(2-tetralonoxy)naphthalene (**22**) 43

1′,5′-Bis((1-methylene-1,2,3,4-tetrahydronaphthalen-2-yl)oxy) naphthalene (**23**) 44

2′,6′-Bis((3,4-dihydronaphthalen-1-yl)methyl) naphthalene-1′,5′-bistrichloroacetate (**25**) 45

7,17-Dichloro-5,6,15,16-tetrahydrodinaphtho [1,2,-b:1',2'-k]chrysene (**26**) 46

7,17-Dichlorodinaphtho[1,2,-b:1',2'-k]chrysene (**27**) 47

7,17-Bis(4-hexylphenyl)dinaphtho[1,2,-b:1',2'-k]chrysene (**28**) 48

6 - XRD Data

6.1 - 4,10-Dichlorochrysene (**5**) 49

6.2 – 4,10-Diphenylchrysene (**6**) 50

6.3 - 4,9-Dichlorobenzo[c]phenanthrene (**11**) 51

6.4 - 4,9-Diphenylbenzo[c]phenanthrene (**12**) 52

6.5 - 7-Chlorobenzo[k]tetraphene (**20**) 53

6.6 - 7-Phenylbenzo[k]tetraphene (**21**) 54

6.7 - 7,17-Dichlorodinaphtho[1,2,-b:1',2'-k]chrysene (**27**) 55

6.8 - 7,17-Bis(4-hexylphenyl)dinaphtho[1,2,-b:1',2'-k]chrysene (**28**) 56

7 – OFET Fabrication 58

1 - General Experimental Procedure

All reactants and reagents were purchased from Sigma-Aldrich (UK) and were used without further purification. Solvents used were purified by standard methods. All reactions, unless otherwise noted, were carried out under N_2 gas using flame-dried glassware. MWI-assisted reactions were performed with a Biotage Initiator microwave reactor. NMR spectra were acquired with B400 Bruker Avance III 400 MHz or B500 Bruker Avance II+ 500 MHz spectrometers, using TMS as an internal standard (0.00 ppm). Mass measurements were acquired with a Micromass Trio 200 spectrometer, using electrospray (ES), atmospheric pressure chemical ionisation (APCI) or matrix-assisted laser desorption ionisation (MALDI) techniques, as stated. High resolution mass spectra were recorded on a Kratos Concept IS spectrometer. Ultraviolet-visible (UV-Vis) spectra were recorded on a Varian Cary 50 spectrophotometer from solutions of DCM. Fluorescence spectra were recorded on a Varian Cary Eclipse spectrophotometer from solutions of DCM. Cyclic Voltammetry (CV) was performed on a BASi-Epsilon platform with a scan rate of 100 mV/s using solutions of 5 - 10 mM of analyte and 100 mM of tetrabutylammonium hexafluorophosphate in DCM.

2 - Synthetic Procedures

2.1 - Preparation of Benzo[c]phenanthrene Derivatives

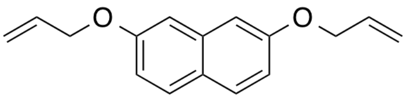

2,7-Bis(allyloxy)naphthalene 8.[1] Allyl bromide (19.45 mL, 224.8 mmol) was added to a solution of potassium carbonate (31.06 g, 224.8 mmol) and 2,7-dihydroxynaphthalene (15 g, 93.7 mmol) in acetone (300 mL) and vigorously stirred at reflux (56 °C) for 30 h. The inorganic solids were removed filtration through celite and the solvent removed *in vacuo*. The solid residue was redissolved in Et_2O (250 mL) and the solution washed with water (2 x 100 mL), NaOH (1 M, 100 mL) and brine (100 mL). The organic phase was dried over $MgSO_4$ and the solvent removed by *in vacuo*. The crude product was purified by recrystallisation from hexane to afford the product 2,7-bis(allyloxynaphthalene) as a white solid (44.24 g, 82%. **MP** 65 – 67 °C. **^{1}H NMR** (500 MHz, Chloroform-d) δ 7.68 (2H, d, H-Ar_4, J = 8.8 Hz), 7.08 (2H, s, H-Ar_1), 7.05 (2H, d, H-Ar_3, J = 8.5 Hz), 6.15 (2H, ddt, C-CH=C, J = 17.3, 10.4, 5.0 Hz), 5.51 (2H, dq, H_Z-C=C, J = 17.3, 1.6 Hz), 5.35 (2H, ddt, H_E-C=C, J = 10.4, 1.3 Hz), 4.67 (4H, 4.67, dt, H_2-C-Ar, J = 5.0, 1.3 Hz) ppm. **^{13}C NMR** (101 MHz, Chloroform-d) ∂ 157.2, 135.8, 133.3, 129.2, 124.5, 117.7, 116.5, 106.5, 68.8 ppm. **MS** (APCI$^+$) m/z 241.1 ([M+H]+, 100%).

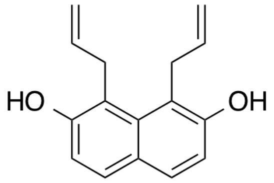

1,8-Diallyl-2,7-dihydroxynaphthalene 9.[1] Bis(allylether) **8** (5 g, 20.81 mmol) was dissolved in pyridine (10 mL) in a 20 mL microwave vial and the solution purged thoroughly with N_2. The vial was then sealed and heated to 160 °C for 8 h in a microwave reactor. After cooling, toluene was added to the solution and the pyridine and toluene azeotrope removed by rotary evaporation. The crude product was then recrystallised from hexane to afford the *title compound* as a red solid (4.00 g, 80%). **MP** 135 °C. **^{1}H NMR** (500 MHz, Chloroform-d) δ 7.46 (2H, d, H-Ar_4, J = 8.8 Hz), 7.00 (2H, d, H-Ar_3, J = 8.8 Hz), 6.25 - 6.32 (2H, m, C-CH=C), 5.12 (2H, dq, H_E-C=, J = 10.4, 2.2 Hz), 4.97 (2H, dq, H_Z-C=, J = 17.3, 1.9 Hz), 3.93 (4H, dt, H_2-C-Ar, *J* = 8.5, 2.5 Hz) ppm. **^{13}C NMR** (101 MHz, Chloroform-d) δ 152.5, 137.3, 126.1, 125.3, 118.9, 117.4, 116.9, 114.7, 39.7 ppm. **MS** ($APCI^+$) m/z 241.2 ([M+H]+, 100%).

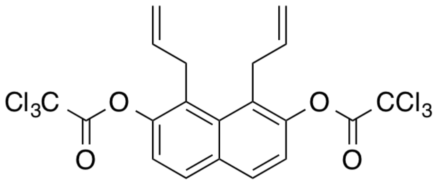

1,8-Diallyl-2,7-bis(2,2,2-trichloroacetyl)naphthalene 10. A solution of 1,8-diallyl-2,7-dihydroxynaphthalene **9** (3 g, 12.48 mmol) and pyridine (2.4 mL, 29.73 mmol) in diethyl ether (20 mL) was cooled to 0 °C. Trichloroacetyl chloride (5.445 mL, 29.73 mmol) was added dropwise to the solution and stirred for 2 h. The reaction was quenched by rapid addition to ice (200 mL), and the organic layer washed quickly with water (1 x 100 mL), sodium hydroxide solution (2 M, 3 x 100 mL) and brine (2 x 100 mL). The product layer was then dried over $MgSO_4$ and the solvent removed by rotary evaporation to afford the *title compound* as a yellow solid (4.31 g, 65%. **MP** 130 °C. **^{1}H NMR** (500 MHz, Chloroform-d) δ 7.93 (2H, d, H-Ar_4, J = 8.8 Hz), 7.60 (2H, d, H-Ar_3, J = 8.8 Hz), 6.12 – 6.22 (2H, m, C-CH=C), 5.18 (2H, dd, H_E-C=C, J = 10.3, 1.3 Hz), 4.84 (2H, dd, H_Z-C=C, J = 17.4, 1.0), 3.92 (4H, dt, H_2-C-Ar, J = 8.3, 2.4 Hz) ppm. **^{13}C NMR** (101 MHz, Chloroform-d) δ 160.6, 148.9, 136.3, 134.3, 132.7, 130.1, 127.1, 120.1, 116.8, 89.6, 31.2 ppm. **MS** ($APCI^+$) m/z 528.9 ([M+H]+, 100%).

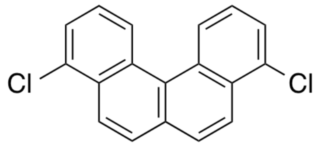

4,9-Dichlorobenzo[c]phenanthrene 11. 1,8-Diallyl-2,7-bis(trichloroacetyl)naphthalene **10** (5 g, 16.82 mmol) and copper (I) chloride (83 mg, 0.841 mmol) were added to a Schlenk tube and the solids dissolved in diglyme (5 mL). The solution was purged thoroughly with N_2 and heated to reflux (162 °C) for 2 h. After cooling, the reaction mixture was diluted with DCM and loaded directly onto a flash chromatography column and eluted with 1:10 DCM/hexane. The product fraction was concentrated by rotary evaporation to afford the crude product as a white solid which was then recrystallised from hexane to afford the *title compound* as white crystals (1.40 g, 28%). **MP** 175 °C. **^{1}H NMR** (400 MHz, Chloroform-d) δ 8.96 (2H, d, H-Ar_1, *J* = 8.7 Hz), 8.48 (2H, d, H-Ar_6, *J* = 8.9 Hz), 7.98 (2H, d, H-Ar_5, *J* = 8.9 Hz), 7.76 (2H, d, H-Ar_3, *J* = 7.7 Hz), 7.61 (2H, apt.t, H-Ar_2, *J* = 8.3 Hz) ppm. **^{13}C NMR** (101 MHz, Chloroform-d) δ 132.6, 131.7, 131.1, 129.9, 127.9, 127.3, 126.7, 126.0, 124.0 ppm. **MS** ($APCI^+$) m/z 297.0 ([M+H]+, 100%). **HRMS** (EI^+) $C_{18}H_{10}Cl_2$ requires 296.0154 found 296.0154.

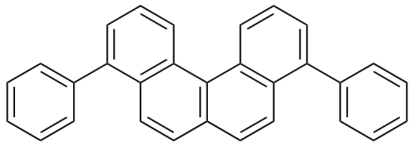

4,9-Diphenylbenzo[c]phenanthrene 12. A solution of phenylmagnesium bromide was prepared by the reaction of bromobenzene (142 μL, 1.35 mmol) and magnesium turnings (65 mg, 2.70 mmol) in THF (1.5 mL). This solution was then transferred by syringe to a stirring solution of 4,9-dichlorobenzo[c]phenanthrene **11** (50 mg, 0.17 mmol) and PEPPSI-IPr (2.25 mg, 3.275 mmol) in THF (1 mL) in a sealable reaction vial. The vial was then capped and heated to 50 °C for 3 h. After cooling, the reaction mixture was loaded directly onto a column of silica and the products eluted with 1:9 DCM/hexane. The homocoupling product biphenyl was then removed from this mixture by trituration with hexane to afford the title compound as a white solid (34 mg, 53 %) A colourless crystal suitable for x-ray crystallography was grown by vdiffusion of hexane vapour into a saturated solution of toluene. **MP** 159 °C. **^{1}H NMR** (400 MHz, Chloroform-d) δ 9.08 (2H, d, H-Ar_1, J = 8.3 Hz), 7.89 (2H, d, H-Ar_6, J = 8.8 Hz), 7.76 – 7.71 (4H, m), 7.64 – 7.48 (12H, m) ppm. **^{13}C NMR** (101 MHz, Chloroform-d) ∂ 142.5, 141.3, 140.8, 139.2, 131.8, 130.7, 130.4, 128.3, 127.9, 127.6, 127.3, 126.5, 126.0, 125.5, 125.3 ppm. **MS** (APCI+) m/z 297.02 ([M+H]+, 100%). **HRMS** (EI^+) $C_{30}H_{20}$ requires 380.1560 found 380.1558.

2.2 - Preparation of Benzo[k]tetraphene Derivatives

CARE: This compound is a potent lachrymator and potential skin vesicant

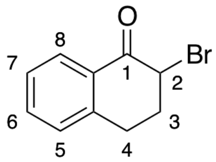

2-Bromo-1-tetralone 14.[2] Bromine (21.89 g, 137 mmol) was added dropwise to a stirring solution of 1-tetralone (20 g, 137 mmol) in sodium dried diethyl ether (100 mL) at 0 °C. After 20 minutes to reaction was quenched by the sequential addition of ice water (100 mL) and aqueous sodium thiosulfate solution (1 M, 100 mL). The ethereal layer was collected, washed with water (2 x 50 mL), brine (50 mL), dried over anhydrous $MgSO_4$ and concentrated *in vacuo* to yield the *title compound* as a brown oil in 88% yield (with 1-tetralone present as a 10% impurity). No further purification was performed due to the lachrymatory nature of the compound. **^{1}H NMR** (500 MHz, Chloroform-*d*) δ 8.10 (1H, dd, H-Ar_8, J = 7.9, 1.2 Hz), 7.53 (1H, td, H-Ar_6, J = 7.5, 1.2 Hz), 7.36 (1H, t, H-Ar_7, J = 7.6 Hz), 7.29 (1H, d, H-Ar_5, J = 7.7 Hz), 4.74 (1H, dd, H-C_2, J = 5.0, 3.5 Hz), 3.32 (1H, ddd, H_2-C_4, J = 17.2, 10.1, 4.7 Hz), 2.93 (1H, dt, H_2-C_4 J = 17.2, 4.4 Hz), 2.60 - 2.39 (2H, m, H_2-C_3) ppm. **^{13}C NMR** (125 MHz, Chloroform-*d*) δ 190.7 (C_1), 143.2, 134.4, 130.1, 129.0, 128.8, 127.3, 50.8 (C_2), 32.1 (C_3), 26.3 (C_4) ppm.

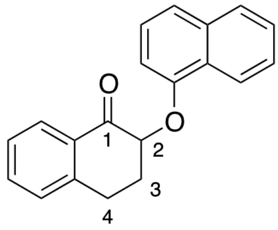

2-(Naphthalene-1′-yloxy)-1-tetralone 15.[3] 2-Bromo-1-tetralone **14** (15.41 g, 68.5 mmol) was added to a stirring suspension of 1-naphthol (9.86 g, 68.5 mmol) and K_2CO_3 (18.91 g, 137 mmol) in dry acetone (200 mL) and the mixture stirred under N_2 for 16 h at ambient temperature. The inorganic materials were then removed by vacuum filtration, the solid was washed with acetone and the solvent removed to afford a brown mass which was then dissolved in Et_2O (200 mL) and washed with NaOH solution (1 M, 5 x 50 mL), water (3 x 50 mL) and brine (50 mL). The organic extract was dried over anhydrous $MgSO_4$ and concentrated *in vacuo* to afford the *title compound* as a brown solid (13.23 g, 67%). **MP** 95 °C. **^{1}H NMR** (400 MHz, Chloroform-*d*) δ 8.38 - 8.31 (1H, m, Ar), 8.09 (1H, dd, Ar, *J* = 7.9, 1.4 Hz), 7.84 - 7.78 (1H, m, Ar), 7.54 (1H, td, Ar, *J* = 7.5, 1.5 Hz), 7.51 - 7.44 (3H, m, Ar), 7.41 - 7.34 (2H, m, Ar), 7.32 (1H, d, Ar, *J* = 7.6 Hz), 6.97 (1H, d, Ar, *J* = 7.7), 5.15 (1H, dd, H-C_2, *J* = 10.4, 5.0 Hz), 3.30 (1H, dt, H_2-C_4, *J* = 17.1, 4.9 Hz), 3.19 (1H, ddd, H_2-C_4, *J* = 17.1, 9.7, 5.3 Hz), 2.69 - 2.53 (2H, m, H_2-C_3) ppm. **^{13}C NMR** (101 MHz, Chloroform-*d*) δ 194.9 (C_1), 153.9 (Np-O), 143.3 (Ar), 134.7 (Ar), 133.9 (Ar), 131.9 (Ar), 128.7 (Ar), 128.0 (Ar), 127.5 (Ar), 127.0 (Ar), 126.4 (Ar), 126.2 (Ar), 125.7 (Ar), 125.4 (Ar), 122.2 (Ar), 121.3 (Ar), 107.5 (Ar), 79.4 (C_2), 30.0 (C_3), 27.5 (C_4) ppm. **MS** (APCI) m/z 289 ([M + H]+, 65%). **HRMS** (ES$^+$) $C_{20}H_{16}O_2Na_1$ requires 311.1043, found 311.1041.

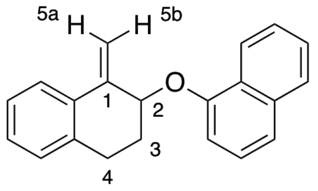

2′-(Naphthalene-1′-yloxy)-1-methylene-2,3,4-trihydronaphthalene 16.
Methyltriphenylphosphonium bromide (28.17 g, 69.4 mmol) and KO^{t}Bu (7.77 g, 69.4 mmol) were suspended in freshly distilled diethyl ether (400 mL) at 0 °C and the resultant turbid yellow mixture allowed to warm to ambient temperature with stirring under N_2. After 20 minutes the solution became clear yellow, signifying consumption of the phosphonium salt. 2-(Naphthalene-1′-yloxy)-1-tetralone **15** (10 g, 34.7 mmol) was suspended in diethyl ether (200 mL) with sonication and the ylide solution transferred to it by cannula. After stirring at ambient temperature for 20 h, the reaction mixture was filtered through celite, silica (50 g) was added and the solvent removed *in vacuo*. The impregnated silica was then eluted through a 10 cm silica plug with hexane, removing elimination byproducts. The silica was then washed with 5% DCM : hexane and the pale yellow solution concentrated *in vacuo* to afford the *title compound* as yellow crystals (10.93 g, 55%). **MP** 101 °C. **^{1}H NMR** (500 MHz, Chloroform-*d*) δ 8.14 (1H, dd, Ar, *J* = 8.4, 1.3 Hz), 7.72 (1H, d, Ar, *J* = 8.1 Hz), 7.56 (1H, dd, Ar, *J* = 7.7, 1.5 Hz), 7.41 - 7.22 (4H, m, Ar), 7.19 - 7.09 (3H, m, Ar), 6.89 (1H, d, Ar, *J* = 7.5 Hz), 5.58 (1H, s, H_a-C_5), 5.29 (1H, s, H_b-C_5), 5.16 (1H, dd, H-C_2, *J* = 8.4, 3.1, Hz), 3.14 (1H, dt, H-C_4, *J* = 17.0, 6.3 Hz), 2.92 (1H, dt, H-C_4, *J* = 17.0, 6.7 Hz), 2.36 - 2.21 (2H, m, H-C_3) ppm. **^{13}C NMR** (125 MHz, Chloroform-*d*) δ 153.7, 142.9, 135.9, 134.7, 133.5, 128.9, 128.0, 127.5, 126.5, 126.3, 126.3, 125.8, 125.2, 125.2, 122.3, 120.6, 110.1, 107.5, 77.2, 29.4, 26.9 ppm. **MS** (APCI) m/z 287 ([M + H]$^+$, 100%). **HRMS** (EI$^+$) $C_{21}H_{18}O_1$ requires 286.1352, found 286.1341.

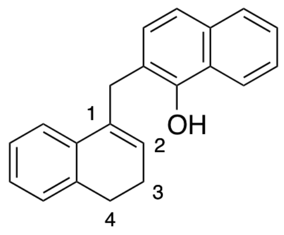

2′-((3,4-Dihydronaphthalen-1-yl)methyl)naphthalen-1′-ol 17. Olefin **16** (2.66 g, 9.38 mmol) and dry pyridine (3 mL) were added to a 5 mL microwave vial, the mixture purged with nitrogen for 20 mins, the vial capped and then heated in a microwave reactor to 115 °C for 2 h. Pyridine was then removed from the product *in vacuo* to yield the pure *title compound* as an orange solid in quantitative yield.

MP 165 °C (decomp.). **^{1}H NMR** (400 MHz, Chloroform-*d,* {appears second order – multiplicities are complex}) δ 8.59 (1H, dt, *J* = 4.2, 1.6 Hz), 8.30 (1H, d, *J* = 8.3 Hz), 7.86 – 7.81 (1H, m), 7.53 – 7.43 (2H, m), 7.41 – 7.08 (5H, m), 5.76 (1H, t, H-C_2, *J* = 4.5 Hz), 3.97 (2H, s, H_2-C-Np), 2.76 (2H, t, H_2-C_4, *J* = 8.23 Hz), 2.27 – 2.20 (2H, m, H_2-C_3) ppm. **^{13}C NMR** (101 MHz, Chloroform-*d*) δ 149.1, 148.7, 136.4, 134.5, 134.3, 133.2, 128.1, 127.2, 127.1, 126.4, 125.9, 125.2, 124.7, 123.7, 122.6, 121.3, 119.3, 32.4 (CH_2-Np), 27.5 (C_4), 22.6 (C_3) ppm. **MS** (APCI) m/z 285 ($[M - H]^-$, 100%).

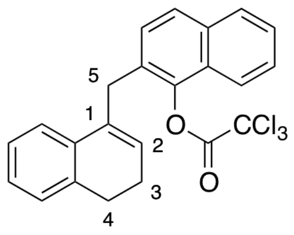

2′-((3,4-Dihydronaphthalen-1-yl)methyl)naphthalen-1′-trichloroacetate 18. Naphthol **17** (2.5 g, 8.74 mmol) and dry pyridine (2.82 mL, 34.96 mmol) were dissolved in dry diethyl ether (100 mL) under nitrogen and the solution cooled to 0 °C. Trichloroacetyl chloride (1.47 mL, 13.11 mmol) was added dropwise with vigorous stirring. After 1 h, solid sodium hydrogen carbonate (10 g) was added and the suspension stirred for 5 mins, after which the inorganics were removed by filtration and the filter cake washed with diethyl ether (100 mL). The ethereal solution was then washed with water (5 x 100 mL) and brine (100 mL), dried over anhydrous $MgSO_4$ and concentrated in vacuo to afford the *title compound* in 40% yield as a colourless oil with 20% of the parent naphthol as an inseperable impurity resulting from hydrolysis of the ester. **MP** 124 °C (decomp.). **^{1}H NMR** (400 MHz, Chloroform-*d*) δ 7.79 (1H, d, Ar, *J* = 8.6 Hz), 7.74 (1H, d, Ar, *J* = 7.5 Hz), 7.61 (1H, d, Ar, *J* = 8.7 Hz), 7.46 (1H, ddd, Ar, *J* = 8.3, 6.9, 1.3 Hz), 7.39 (1H, ddd, Ar, *J* = 8.1, 6.9, 1.3 Hz), 7.27 (1H, d, Ar, *J* = 8.5 Hz), 7.09 - 6.98 (4H, m, Ar), 5.70 (1H, t, H-2, *J* = 4.5 Hz), 3.79 (2H, s, H_2-5), 2.69 (2H, t, H-4, *J* = 8.0 Hz), 2.20 (2H, H-3, m). **^{13}C NMR** (101 MHz, Chloroform-*d*) δ 160.3 (C=O), 143.8 (Np-O), 136.6, 134.6, 133.6, 133.3, 128.6, 128.5, 128.1, 127.6, 127.5, 127.3, 127.3, 127.0, 126.6, 126.3, 122.9, 120.3, 89.8 (CCl_3), 33.0 (C_5), 28.3 (C_4), 23.3 (C_3). **MS** (APCI) m/z 431 ([M{$^{35}Cl_3$} + H]$^+$, 15%), 433 ([M{$^{35}Cl_2$ + ^{37}Cl} + H]$^+$, 8%). **HRMS** (EI$^+$) $C_{23}H_{17}Cl_3O_2$ requires 394.0522, found 394.0522.

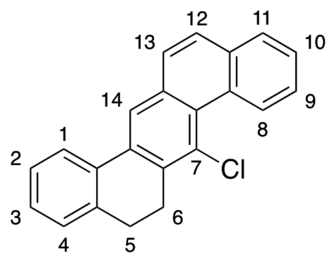

7-Chloro-5,6-dihydrobenzo[k]tetraphene 19. Trichloroacetate **18** (1.90 g, 4.40 mmol) and cuprous chloride (5 mol%, 22 mg, 220 μmol) were dissolved in dry diglyme (2 mL), the solution purged with nitrogen for 20 mins and then heated to reflux (162 °C) for 2 h. After cooling, the product was isolated by column chromatography (1:9 DCM:hexane) to yield the *title compound* as a white solid (693 mg, 50%). **MP** 133 °C. **^{1}H NMR** (500 MHz, Chloroform-*d*) δ 9.90 (1H, d, H-Ar_8, J = 8.2 Hz), 8.16 (1H, s, H-Ar_{14}), 7.91 (1H, d, J = 7.8 Hz), 7.88 – 7.86 (1H, m), 7.70 (2H, s, H-Ar_{12}, H-Ar_{13}), 7.65 – 7.58 (2H, m), 7.39 – 7.34 (1H, m), 7.32 – 7.29 (2H, m), 3.36 (2H, dd, H_2-C_6, J = 7.3, 5.4 Hz), 2.98 (2H, dd, H_2-C_5, J = 7.3, 5.4 Hz) ppm. **^{13}C NMR** (125 MHz, Chloroform-*d*) δ 137.5, 136.2, 134.0, 133.8, 133.7, 133.5, 130.7, 129.8, 128.6, 128.1, 127.9, 127.7 (C_8), 127.2, 126.7, 125.7, 124.5, 122.8 (C_{14}), 27.07 (C_5), 28.70 (C_6) ppm. **MS** (EI^+) m/z 314 ($[M\{^{35}Cl\}]^+$, 100%), 316 ($[M\{^{37}Cl\}]^+$, 35%). **HRMS** (EI^+) $C_{22}H_{15}Cl_1$ requires 314.0865, found 314.0857.

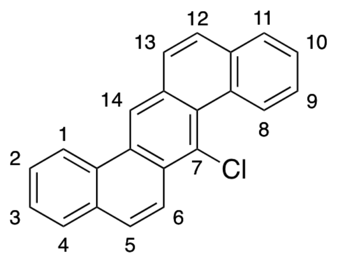

7-Chlorobenzo[*k*]tetraphene 20. 2,3-Dichloro-5,6-dicyano-1,4-benzoquinone (DDQ) (994 mg, 4.38 mmol) was added to a solution of compound **19** (690 mg, 2.19 mmol) in *o*-DCB (5 mL) and heated to 150 °C for 30 min. After cooling, the reaction was diluted with DCM (100 mL) and quenched by the addition of water (25 mL). The aqueous fraction was removed, the organic solution washed with $NaHCO_3$ solution (2 x 50 mL), water (2 x 50 mL) and brine (50 mL) and the solvent removed *in vacuo*. The crude product was then purified by column chromatography with 20% DCM:hexane affording the *title compound* as an white solid (445 mg, 65%). Large colourless crystals for x-ray diffraction analysis were grown by vapour diffusion of toluene/hexane. **MP** 181 °C. **^{1}H NMR** (400 MHz, Chloroform-*d*) δ 9.88 (1H, dd, H-Ar_8, J = 8.0, 1.7 Hz), 8.95 (1H, s, H-Ar_{14}), 8.73 (1H, d, J = 8.1 Hz), 8.52 (1H, d, J = 9.4 Hz), 7.86 - 7.72 (4H, m), 7.67 - 7.50 (5H, m) ppm. **^{13}C NMR** (101 MHz, Chloroform-*d*) δ 133.8, 132.4, 131.6, 129.8, 129.7, 129.6, 129.4, 128.6, 128.6, 128.2, 128.1, 127.9, 127.7, 127.6, 127.3, 127.3, 127.2, 126.6, 125.8, 123.7, 123.19, 121.83. **MS** (EI$^+$) m/z 312 ([M{^{35}Cl}]$^+$, 100%), 314 ([M{^{37}Cl}]$^+$, 35%). **HRMS** (EI$^+$) $C_{22}H_{13}Cl_1$ requires 312.0700 found 312.0700.

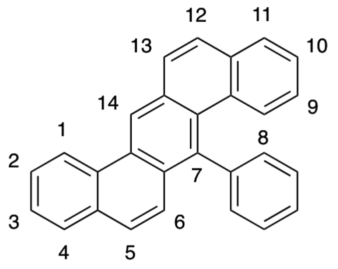

7-Phenylbenzo[*k*]tetraphene 21. A solution of phenylmagnesium bromide (120 μmol) in THF (2 mL) was added to a stirring solution of 7-chlorobenzo[*k*]tetraphene **20** (25 mg, 80μmol) and PEPPSI-IPr (5 mol%, 3 mg, 4 μmol) in THF (2 mL) at ambient temperature. After stirring under nitrogen for 1 h, silica (2.5 g) was added directly to the reaction mixture and the solvent removed *in vacuo*. The impregnated silica was then loaded onto a column and the product purified by chromatography (15% DCM/hexane) to afford the *title compound* as a white solid (20 mg, 71%). A colourless cubic crystal suitable for x-ray analysis was grown by the slow evaporation of chloroform. **MP** 202 °C. **^{1}H NMR** (500 MHz, Chloroform-*d*) δ 9.23 (1H, s, H-Ar_{14}), 8.92 (1H, d, *J* = 8.3 Hz), 7.97 (1H, d, *J* = 8.9 Hz), 7.85 (1H, d, *J* = 7.9 Hz), 7.81 (1H, d, *J* = 7.9 Hz), 7.74 – 7.70 (2H, m), 7.66 – 7.59 (4H, m), 7.57 (1H, d , *J* = 9.5 Hz), 7.50 (1H, d, *J* = 9.5 Hz), 7.49 (1H, d, *J* = 9.0 Hz), 7.45 – 7.41 (3H, m), 7.09 (1H, ddd, *J* = 8.6, 7.0, 1.5 Hz, H-Ph_{para}) ppm. **^{13}C NMR** (125 MHz, Chloroform-*d*) δ 143.1, 138.2, 134.1, 131.9, 131.7, 131.2, 131.0, 130.8, 130.2, 129.9, 129.8, 128.8, 128.7, 128.6, 128.5, 128.1, 127.9, 127.8, 127.4, 127.1, 126.4, 125.8, 125.7, 123.4, 123.1 ppm. **MS** (MALDI-TCNQ$^+$) m/z 354 ([M]$^+$, 100%). **HRMS** (EI$^+$) $C_{28}H_{18}$ requires 354.1403 found 354.1398.

2.3 – Preparation of Dinaphtho[1,2,-*b*:1',2'-*k*]chrysene Derivatives

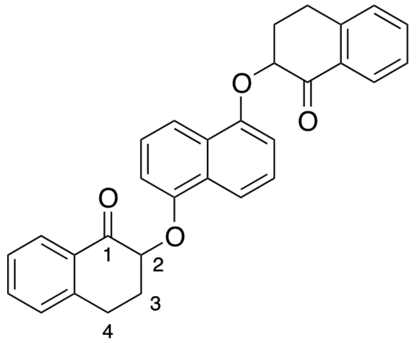

1,5-Bis(2-tetralonoxy)naphthalene 22.[4] 2-Bromotetralone **13** (30.70 g, 136.4 mmol) was added to a stirring suspension of 1,5-dihydroxynaphthalene (7.64 g, 47.7 mmol) and potassium carbonate (18.82 g, 136.4 mmol) in dry DMF (100 mL) and heated to 50 °C under nitrogen for 24 h. The reaction mixture was then diluted with diethyl ether (900 mL) and the precipitate collected by vacuum filtration. The filter cake was then washed with water (1 L) to afford a black solid which was then suspended in hot acetone (200 mL) and the product precipitated with cold methanol which was then collected by filtration. The *title compound* was isolated as a brown solid (6.63 g, 31%). **MP** 140 °C. **^{1}H NMR** (400 MHz, DMSO-d_6) δ 7.91 (2H, d, Ar, *J* = 7.2 Hz), 7.81 (2H, d, Ar, *J* = 8.5 Hz), 7.64 (2H, t, Ar, *J* = 7.5 Hz), 7.48 – 7.35 (6H, Ar, m), 7.12 (2H, d, Ar, *J* = 7.7 Hz), 5.59 (2H, dd, H-C_2, *J* = 12.2, 4.6 Hz), 3.40 – 3.29 (2H, m, H-C_4), 3.18 (2H, dt, H-C_4, *J* = 17.5, 4.1 Hz), 2.64 – 2.56 (2H, m, H-C_3), 2.49 – 2.36 (2H, m, H-C_3) ppm (splitting of H-C_4 due to diastereomers). **^{13}C NMR** (101 MHz, Chloroform-*d*) δ 194.8 (C_1), 153.8 (C_{Np}-O), 143.3, 133.8, 131.9, 128.7, 128.0, 127.50, 126.9, 125.1, 115.7, 108.3, 79.5 (C_2), 30.0 (C_3), 27.4 (C_4). **MS** (MALDI-Dithranol$^+$) m/z 448 ([M+H]$^+$, 100%). **HRMS** (EI$^+$) $C_{30}H_{25}O_4$ requires 449.1753 found 449.1755.

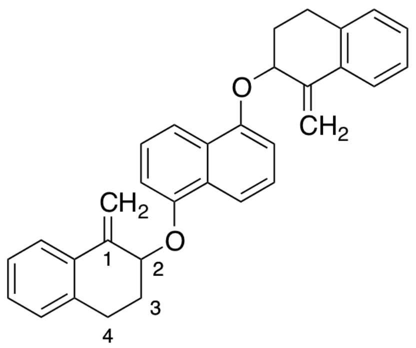

1′,5′-Bis((1-methylene-1,2,3,4-tetrahydronaphthalen-2-yl)oxy)naphthalene 23. Methyltriphenylphosphonium iodide (9.88 g, 24.34 mmol) and potassium *tert*-butoxide were suspended in dry THF (100 mL) and the yellow mixture stirred under nitrogen for 10 minutes. The ylide solution was then transferred to a suspension of compound **22** (4.20 g, 9.36 mmol) in THF (100 mL) and the resultant brown mixture stirred at ambient temperature for 44 h. The reaction mixture was then diluted with hexane (600 mL), the insoluble components removed by filtration and the organic solution washed with water (2 x 300 mL), brine (150 mL), dried over $MgSO_4$ and concentrated *in vacuo*. Triphenylphosphine oxide was removed from this crude product by repeated trituration with methanol (5 x 10 mL) to afford the *title compound* as a cream solid (2.50 g, 60%). **MP** 200 °C. **^{1}H NMR** (400 MHz, Chloroform-*d*) δ 7.73 (2H, d, J = 8.6 Hz), 7.54 (2H, dd, J = 7.7, 1.3 Hz), 7.21 (2H, dd, J = 8.5, 7.6 Hz), 7.16 – 7.06 (6H, m), 6.88 (2H, d, J = 7.6 Hz), 5.56 (2H, s, H_2-C=C), 5.27 (2H, s, H_2-C=C), 5.11 (2H, dd, H-C_2, J = 8.0, 3.4 Hz), 3.11 (2H, dt, H-C_4, J = 17.2, 6.2 Hz), 2.89 (2H, dt, H-C_4, J = 17.1, 6.9 Hz), 2.33 - 2.18 (4H, m, H-C_3) ppm (splitting of H-C_4 due to diastereomers). **^{13}C NMR** (101 MHz, Chloroform-*d*) δ 153.5 (C_{Np}-O), 142.9, 135.9, 133.6, 128.9, 128.0, 127.8, 126.3, 125.2, 125.0, 115.0, 110.1, 108.3, 77.2 (C_2), 41.4 (C_3), 29.1 (C_4) ppm. **MS** (APCI$^+$) m/z 445 ([M+H]$^+$, 100%). **HRMS** (EI$^+$) $C_{32}H_{29}O_2$ requires 445.2168 found 445.2166.

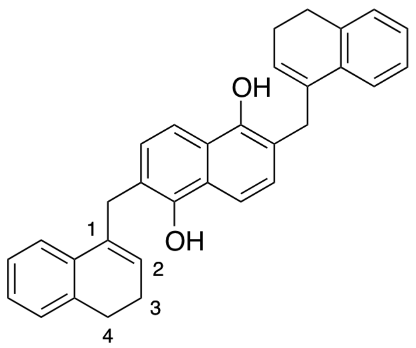

2′,6′-Bis((3,4-dihydronaphthalen-1-yl)methyl)naphthalene-1′,5′-diol 24.

Compound **23** (3.10 g, 6.97 mmol) and dry pyridine (6 mL) were added to a 10 mL microwave vial, the mixture purged with nitrogen for 20 mins, the vial capped and then heated in a microwave reactor to 115 °C for 2 h. Attempts to completely isolate diol **178** from pyridine resulted in its complete decomposition - ^{1}H NMR acquired as 1:1 mixture with pyridine. **^{1}H NMR** (400 MHz, Chloroform-*d*) δ 7.73 (2H, d, H-$Np_{4,}$ J = 8.4 Hz), 7.37 (2H, m), 7.31 (2H, d, H-Np_3, J = 8.4 Hz), 7.20 – 7.15 (6H, m), 5.92 (2H, t, H-C_2, J = 4.5 Hz), 5.67 (2H, bs, OH) 3.67 (4H, s, Ar-**CH_2**-C=), 2.82 (4H, t, H-C_4, J = 8.3 Hz), 2.38 – 2.27 (4H, m, H-C_3) ppm. **MS** ($APCI^+$) m/z 445 ($[M+H]^+$, 100%), 446 ($[M\{^{13}C\}+H]^+$, 35%). **HRMS** (ES^+) $C_{32}H_{29}O_2$ requires 445.2168 found 445.2152.

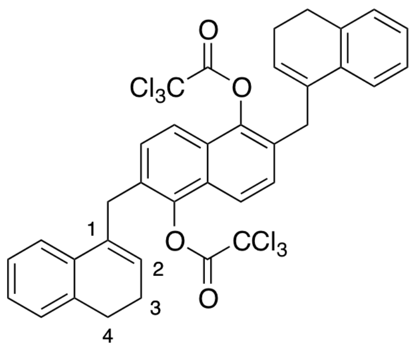

2′,6′-Bis((3,4-dihydronaphthalen-1-yl)methyl)naphthalene-1′,5′-bistrichloroacetate 25. Compound **24** (3.00 g, 6.75 mmol) and dry pyridine (5.80 mL, 72 mmol) were dissolved in dry DCM (50 mL) under nitrogen and the solution cooled to 0 °C. Trichloroacetyl chloride (2.27 mL, 20.25 mmol) was added dropwise with vigorous stirring. The mixture was stirred for 30 min, after which the reaction was quenched by the addition of ice-water (100 mL), diluted with DCM (200 mL) and the organic fraction washed with water (5 x 100 mL). The solution was then concentrated *in vacuo*. Toluene (100 mL) was added to aid azeotropic evaporation of the remaining pyridine. The *title compound* was produced as a golden yellow solid (4.52 g, 91%) and required no further purification. **MP** 211 °C (decomp.). ^{1}**H NMR** (400 MHz, Chloroform-*d*) δ 7.78 (2H, d, J = 8.7 Hz), 7.46 (2H, d, J = 8.7 Hz), 7.29 - 7.23 (2H, m), 7.21 - 7.09 (6H, m), 5.84 (2H, t, H-C_2, J = 4.7 Hz), 3.88 (4H, s, Ar-**CH_2**-C=), 2.81 (4H, t, H_2-C_4, J = 8.0 Hz), 2.36 - 2.27 (4H, m, H_2-C_3) ppm. ^{13}**C NMR** (101 MHz, Chloroform-*d*) δ 160.1 (C=O), 143.8 (C_{Np}-O), 136.5, 134.3, 133.0, 129.4, 128.9, 127.6, 127.1, 126.6, 126.6, 122.8, 119.7, 89.5 (CCl_3), 32.8 (CH_2-Np), 28.2 (C_3), 23.3 (C_4) ppm. **MS** (MALDI-Dithranol$^+$) m/z 757 ($[M+Na]^+$, 100%). **HRMS** (EI^+) $C_{28}H_{18}$ requires 354.1403 found 354.1398. **HRMS** (EI^+) $C_{36}H_{27}O_4Cl_6$ requires 733.0041 found 733.0027.

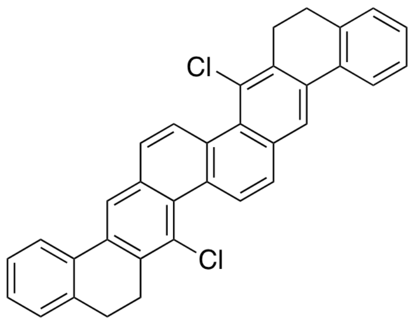

7,17-Dichloro-5,6,15,16-tetrahydrodinaphtho[1,2,-*b*:1',2'-*k*]chrysene 26. Trichloroacetate **25** (500 mg, 0.68 mmol) and copper-NHC **29** (17 mg, 34 μmol) were dissolved in 1,2-dichloroethane (5 mL) in a 10 mL microwave vial and the solution purged with nitrogen for 20 minutes. The vial was then sealed and heated to 200 °C for 2 h. After cooling, the reaction mixture was diluted with ethanol (200 mL), cooled to 0 °C and the precipitate collected by vacuum filtration. The brown solid was washed with water and cold ethanol to afford the *title compound* (297 mg, 87%). **MP** 242-245 °C. **^{1}H NMR** (400 MHz, Chloroform-*d*) δ 9.26 (2H, d, H-C_8, *J* = 9.1 Hz), 8.18 (2H, H-C_{10}, s), 7.90 (2H, d, *J* = 7.6 Hz), 7.75 (2H, d, H-C_9, *J* = 9.1 Hz), 7.34 (2H, dt, *J* = 8.0, 4.3 Hz), 7.27 (4H, d, *J* = 4.0 Hz), 3.32 (4H, dd, *J* = 8.3, 5.8 Hz), 2.95 (4H, dd, *J* = 8.3, 5.8 Hz) ppm. **^{13}C NMR** (101 MHz, Chloroform-*d*) δ 137.6, 136.0, 134.3, 133.7, 133.0, 130.3, 130.0, 128.2, 128.1, 127.5, 127.2, 126.7 (C_8), 124.6, 124.4 (C_9), 121.7 (C_{10}), 28.7, 27.1 ppm. **MS** (MALDI-Dithranol$^+$) m/z 501 ([M+H]$^+$, 100%). **HRMS** (EI$^+$) $C_{34}H_{23}Cl_2$ requires 501.1177 found 501.1176.

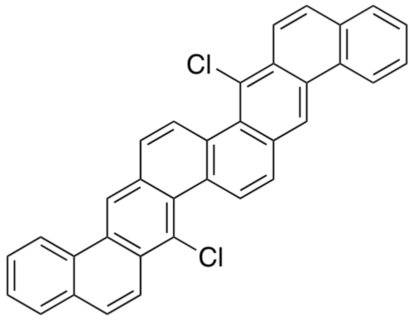

7,17-Dichlorodinaphtho[1,2,-*b*:1',2'-*k*]chrysene 27. Compound **26** (150 mg, 299 μmol), 2,3-dichloro-5,6-dicyano-1,4-benzoquinone (DDQ) (204 mg, 897 μmol) and 1,2-dichlorobenzene (10 mL) were added to a 20 mL microwave vial, the solution purged with nitrogen for 20 minutes, the vial sealed and heated to 150 °C for 30 min in a microwave reactor. After cooling, the mixture was diluted with DCM (100 mL) and the precipitate collected by vacuum filtration. The filter cake was then washed with water (50 mL), methanol (50 mL), acetone (50 mL) and DCM (50 mL) to afford the *title compound* (122 mg, 83%) as an insoluble fine brown solid. Crystallisation from boiling 1,2-dichlorobenzene afforded small golden crystals suitable for x-ray diffraction analysis. **MP** >350 °C. **^{1}H NMR** (500 MHz, DMSO-d_6) δ 9.70 (2H, s, H-CAr$_{10}$), 9.41 (2H, d, J = 9.2 Hz), 9.15 (2H, d, J = 8.3 Hz), 8.57 (2H, d, J = 9.4 Hz), 8.32 (2H, d, J = 9.3 Hz), 8.13 (4H, d, J = 9.0 Hz), 7.88 (2H, t, J = 7.3 Hz), 7.82 (2H, t, J = 7.2 Hz) ppm. **MS** (MALDI-DCTB) m/z 461 ([M-Cl]$^+$, 30%), 495 ([M-H]$^+$, 100%), 529 ([M+Cl-2H]$^+$, 20%). **HRMS** (EI$^+$) $C_{34}H_{18}Cl_2$ requires 496.0780 found 496.0759.

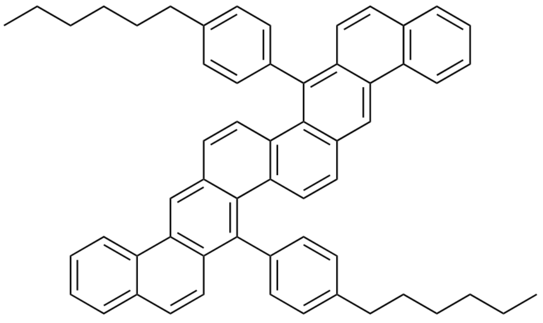

7,17-Bis(4-hexylphenyl)dinaphtho[1,2,-*b*:1',2'-*k*]chrysene 28. 4-Bromo-1-hexylbenzene (408 μL, 2 mmol) was added to a stirring mixture of magnesium turnings (96 mg, 4 mmol) in THF (2.5 mL). The magnesium was activated mechanically with a glass rod, after which the reaction warmed, became turbid, then became a clear brown colour. After the Grignard solution has cooled, it was transferred by syringe to a stirring suspension of 7,17-dichlorodinaphtho[1,2,-*b*:1',2'-*k*]chrysene **27** (100 mg, 201 μmol) and PEPPSI-iPr (14 mg, 20 μmol) in THF (10 mL) and the reaction mixture stirred at room temperature. After 20 h, the mixture was diluted with Et_2O (50 mL), filtered through celite and the solvent removed *in vacuo*. The solid residue was then triturated with MeOH (50 mL) and hexane (50 mL) with sonication, the solid collected and purified further by column chromatography (1:9 DCM:hexane) to afford the title compound as a fluorescent yellow solid (150 mg, 88 %). **MP** 254 °C. **^{1}H NMR** (400 MHz, Chloroform-d) δ 9.06 (2H, s), 8.79 (2H, d, *J* = 8.2 Hz), 7.79 (2H, d, *J* = 7.58 Hz), 7.60 - 7.69 (6H, m), 7.56 (2H, t, *J* = 7.20 Hz), 7.51 (4H, d, *J* = 9.41 Hz), 7.34 (8H, s), 2.76 (4H, t, *J* = 7.58 Hz), 1.75 (4H, tt, *J* = 7.30, 7.27 Hz), 1.34 - 1.46 (12H, m), 0.93 (6H, t, *J* = 6.60 Hz) ppm. **^{13}C NMR** (101 MHz, Chloroform-*d*) δ 142.2, 139.5, 137.9, 131.5, 131.4, 131.4, 130.9, 130.2, 129.9, 129.3, 128.8, 128.6, 128.3, 127.4, 127.1, 126.8, 126.7, 125.8, 124.8, 123.2, 121.5, 35.9, 31.9, 31.6, 29.0, 22.8, 14.3 ppm. **MS** (MALDI-dithranol) m/z 749 ($[M+H]^+$, 100%). **HRMS** (EI^+) $C_{58}H_{52}$ requires 749.4142 found 749.4129.

References

1. Majumdar, K. C.; Chattopadhyay, B.; Chakravorty, S. *Synthesis*, **2009**, 674-680.
2. Barfknecht, C. F.; Rusterholz, D. B.; Parson, J. A. *J. Med. Chem.* **1974**, *17*, 308-3012.
3. Dirania, M. K. M., *Chem. Ind. (London)* **1975**, 926.
4. Jørgensen, M.; Krebs, F.; Bechgaard, *J. Org. Chem.* **2000**, *65*, 8783-8785.

3 – Absorption and Fluorescence Spectra of Key Compounds

Black – UV/vis absorption trace **Red** – Fluorescence emission trace

3.1 - 4,10-Dichlorochrysene (5)

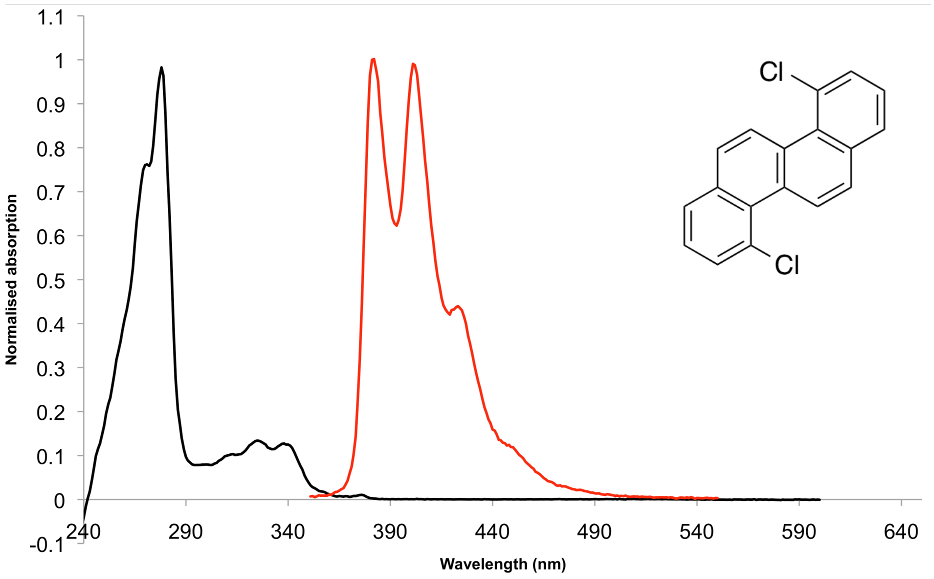

3.2 - 4,10-Diphenylchrysene (6)

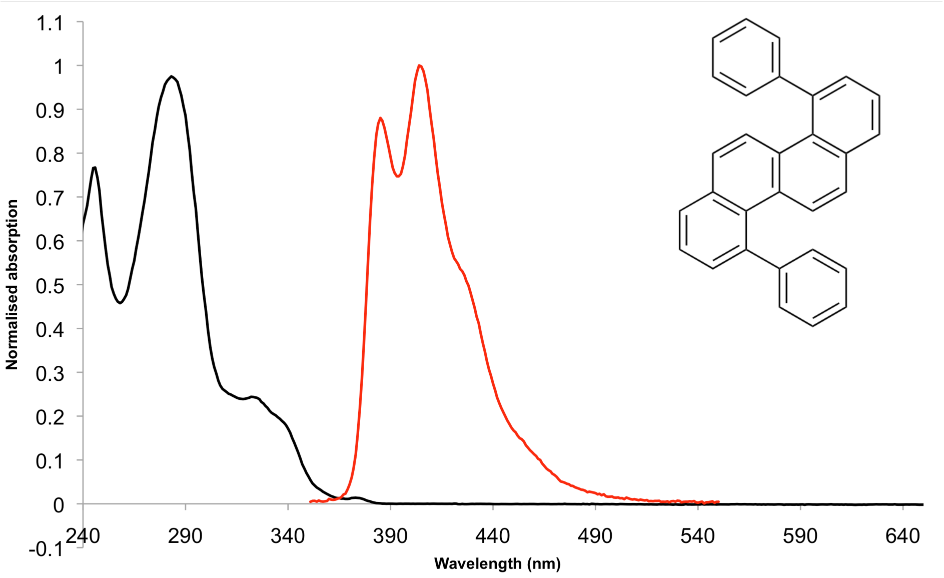

3.3 - 4,9-Dichlorobenzo[c]phenanthrene (11)

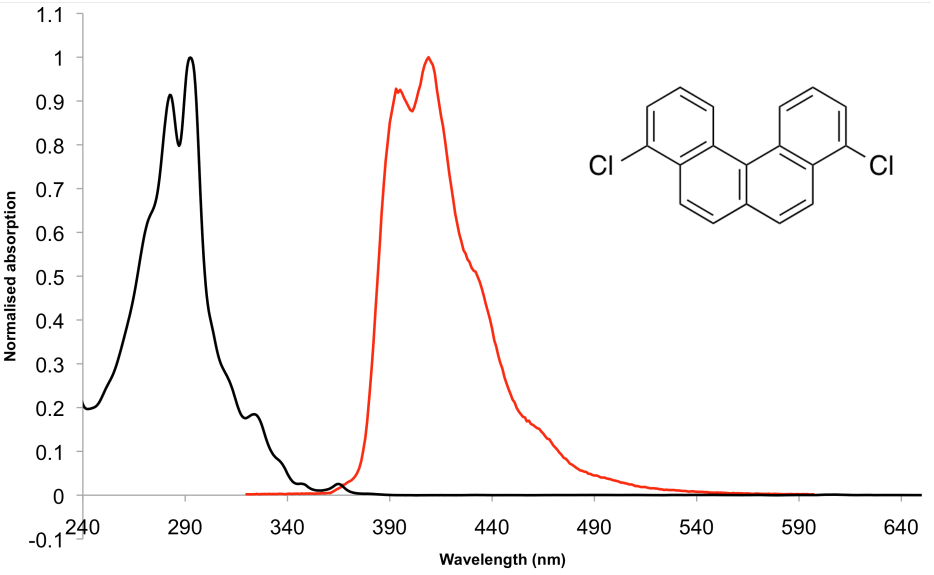

3.4 - 4,9-Diphenylbenzo[c]phenanthrene (12)

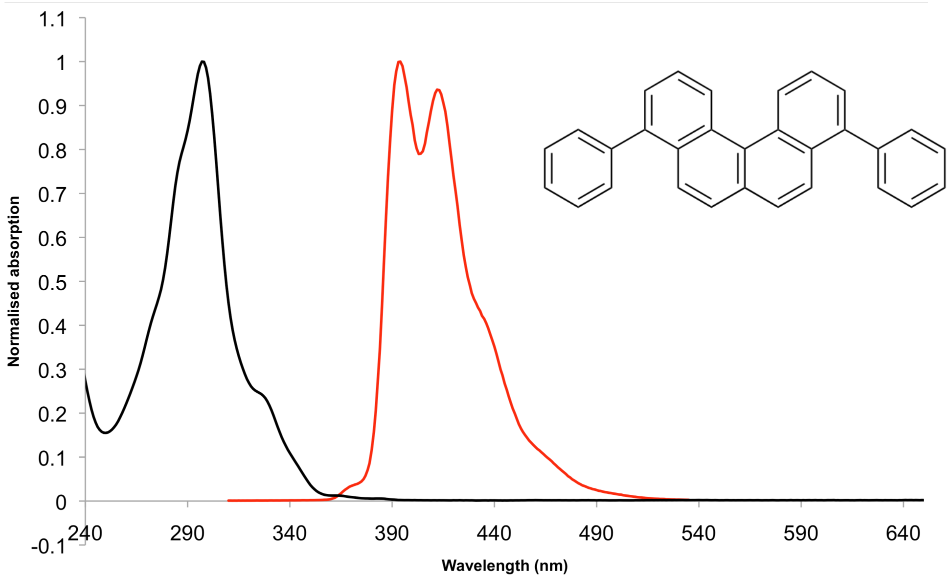

3.5 - 7-Chlorobenzo[k]tetraphene (20)

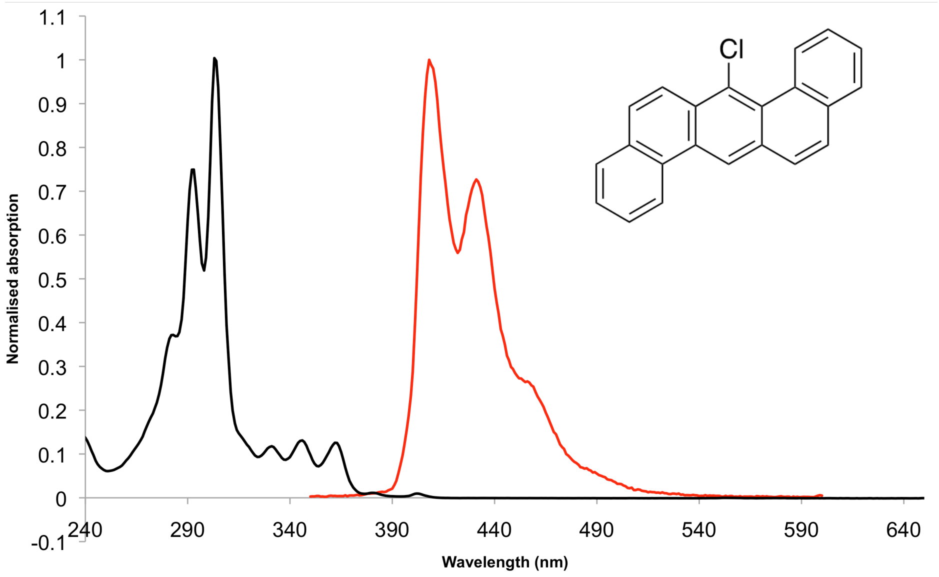

3.6 - 7-Phenylbenzo[k]tetraphene (21)

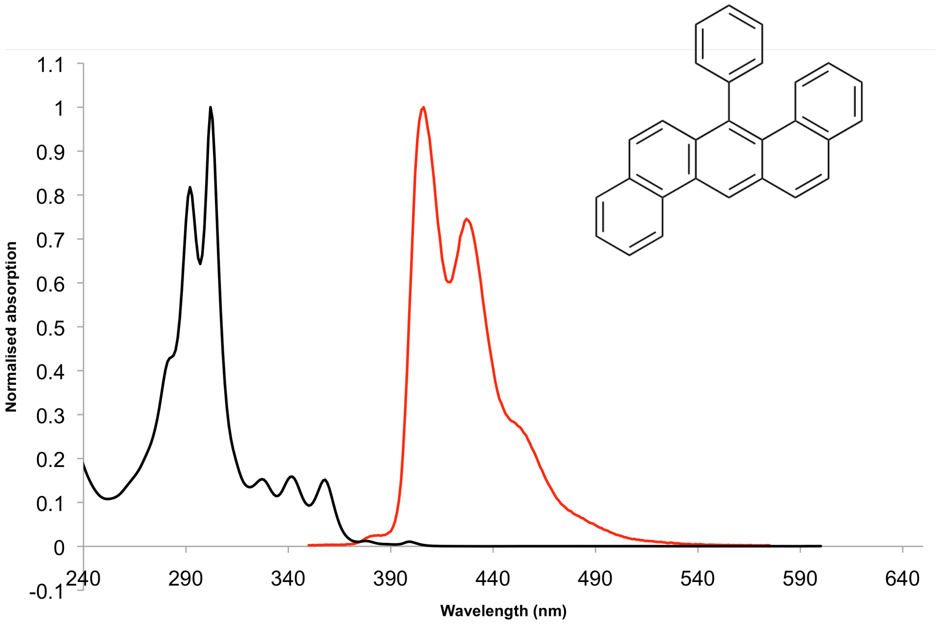

3.7 – 7,17-Dichlorodinaphtho[1,2,-*b*:1',2'-*k*]chrysene (27)

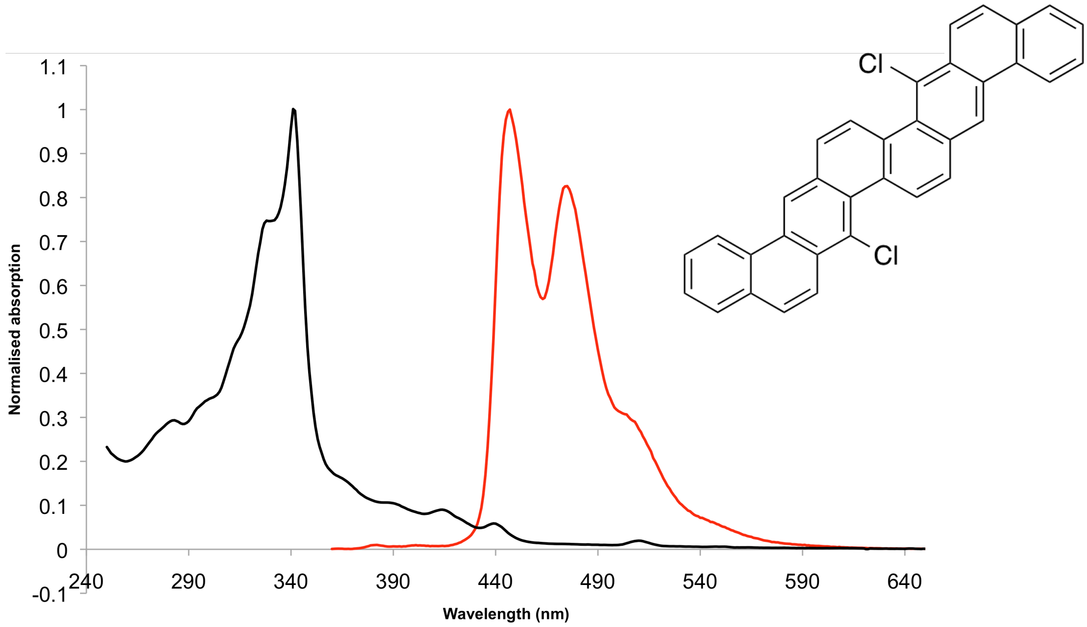

3.8 – 7,17-Bis(4-hexylphenyl)dinaphtho[1,2,-*b*:1',2'-*k*]chrysene (28)

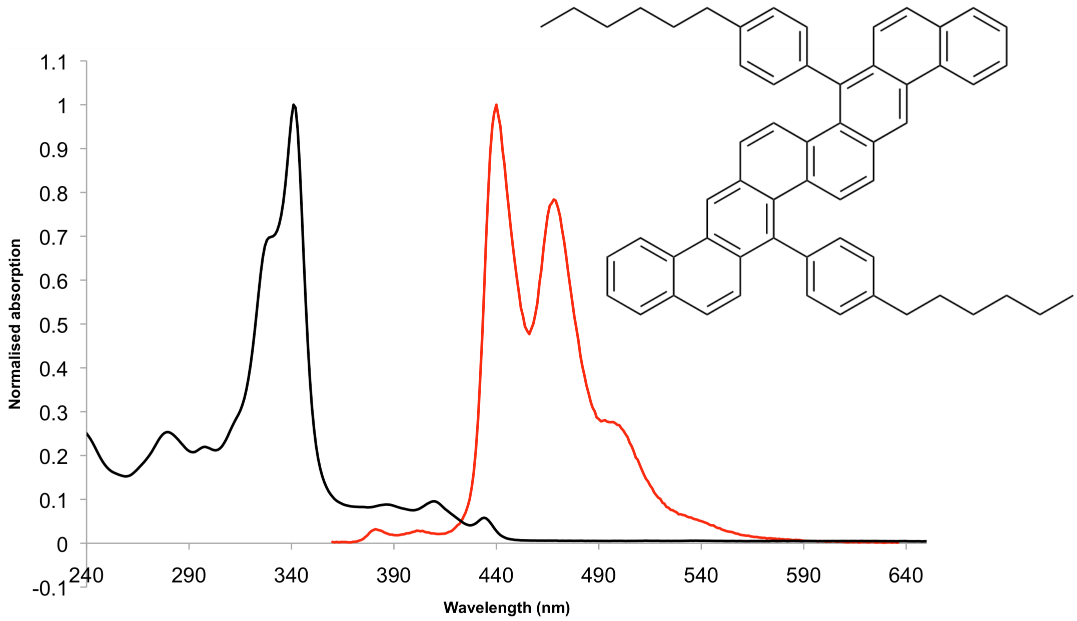

4 – Cyclic Voltammetry of Key Compounds

Black – CV trace of analyte   **Red** – CV trace with ferrocene reference added

4.1 - 4,10-Dichlorochrysene (5)

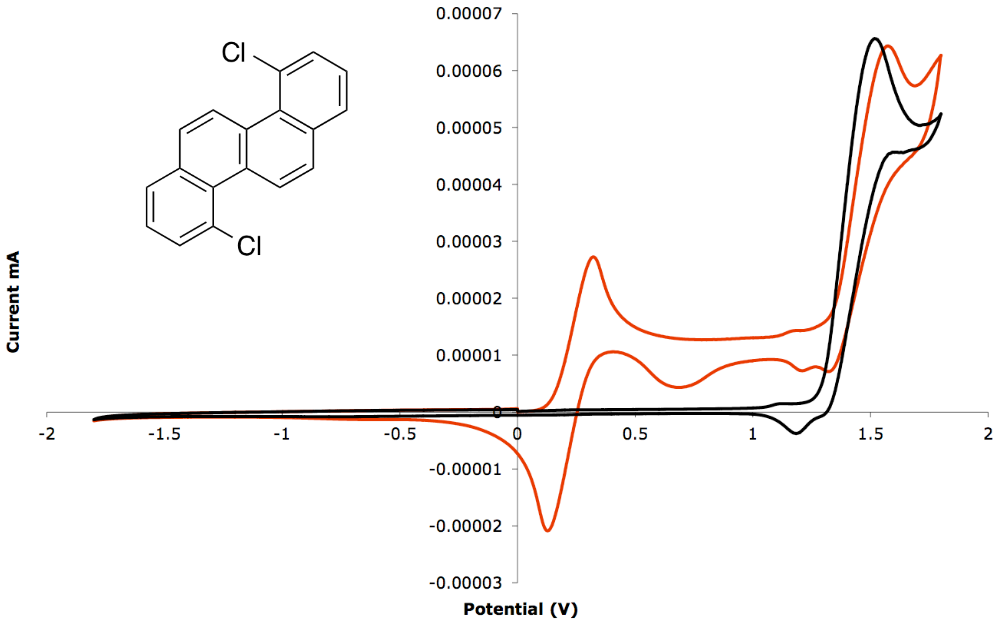

4.2 - 4,10-Diphenylchrysene (6)

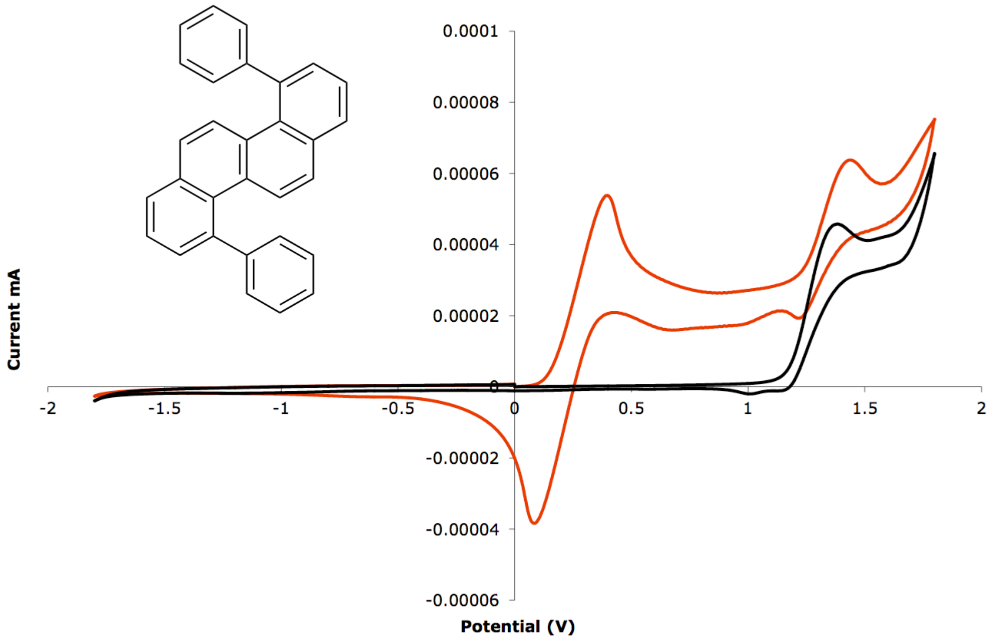

4.3 - 4,9-Dichlorobenzo[c]phenanthrene (11)

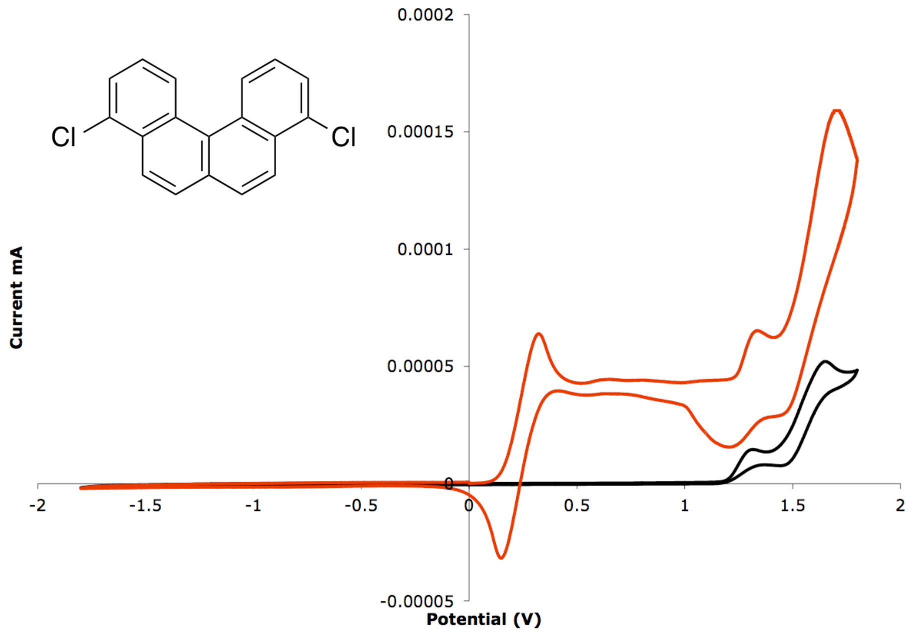

4.4 - 4,9-Diphenylbenzo[c]phenanthrene (12)

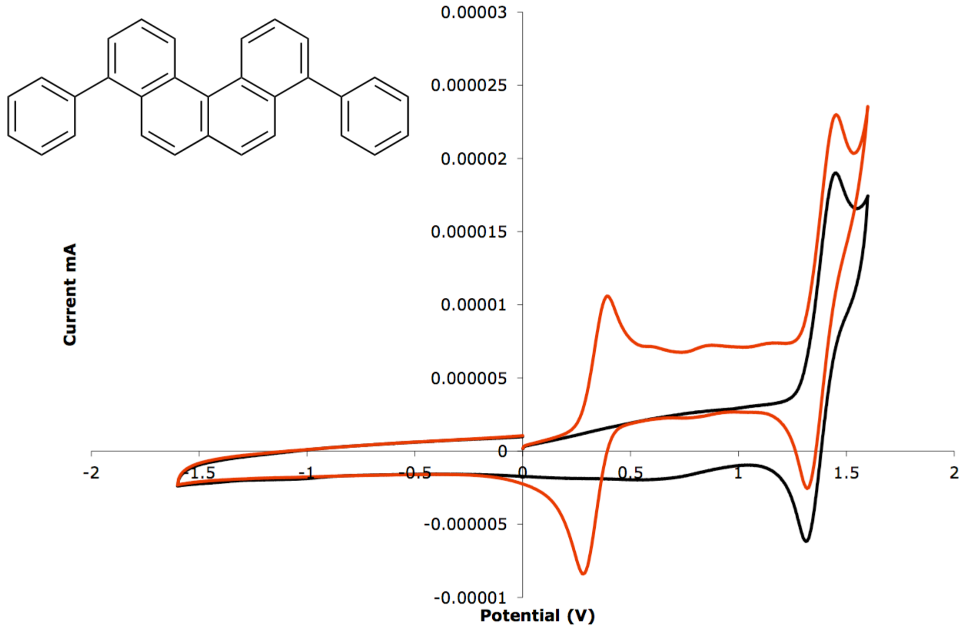

4.5 - 7-Chlorobenzo[k]tetraphene (20)

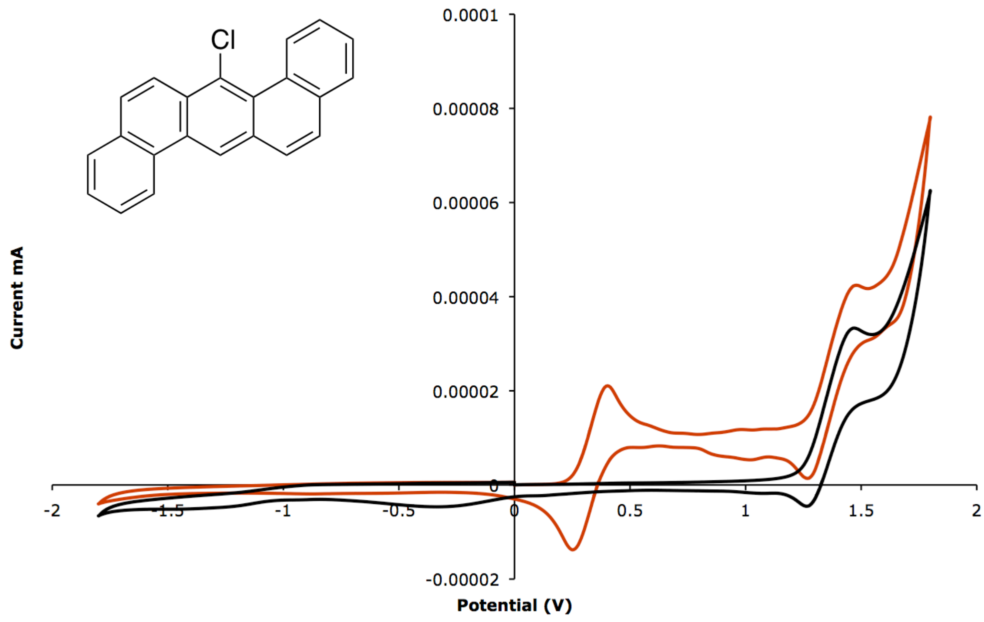

4.6 - 7-Phenylbenzo[k]tetraphene (21)

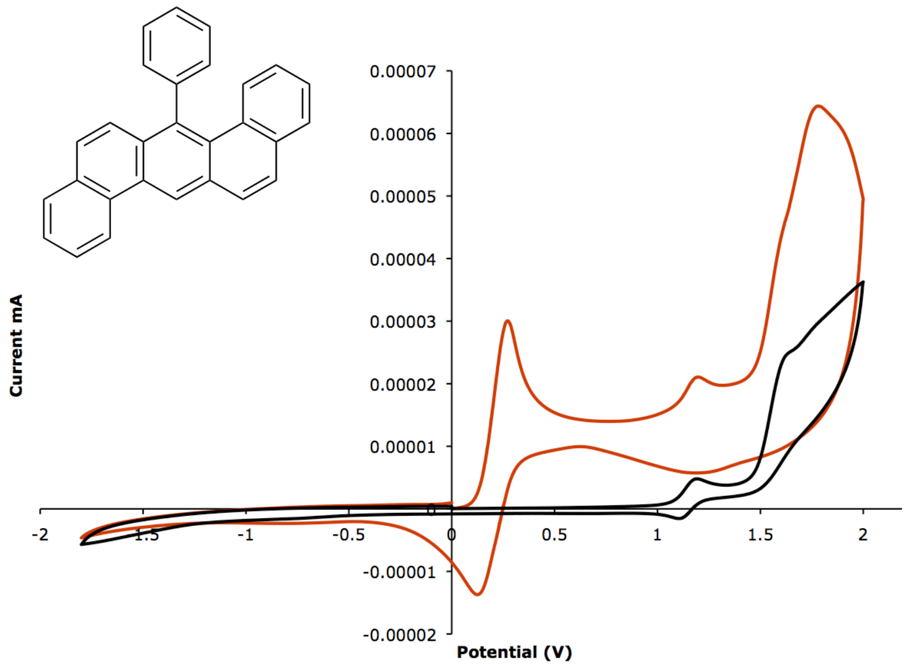

4.7 – 7,17-Dichlorodinaphtho[1,2,-*b*:1',2'-*k*]chrysene (27)

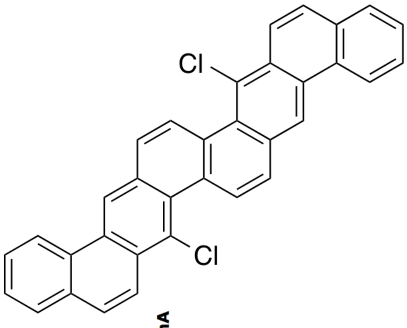

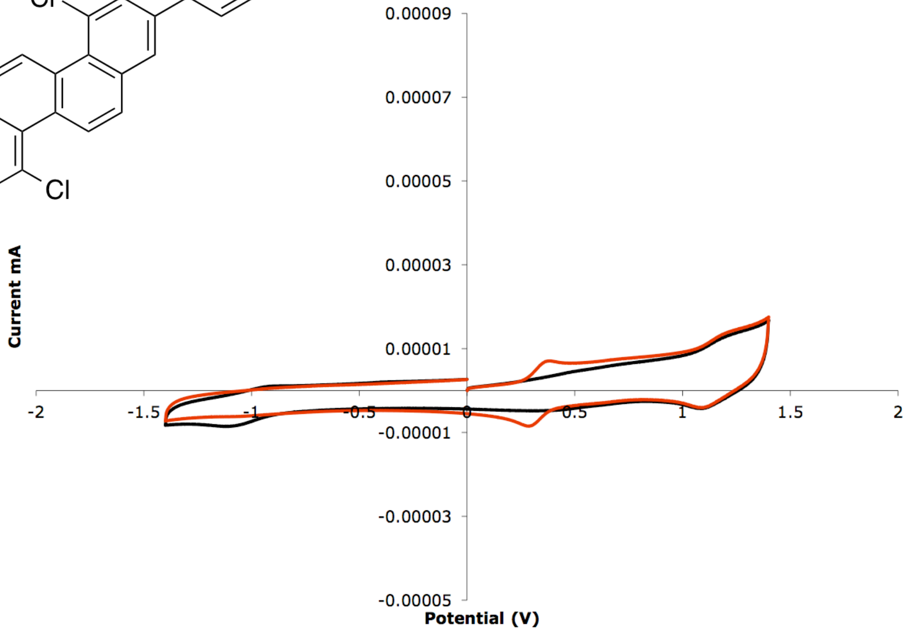

The poor solubility of **27** results in very low CV currents – as such the oxidation peak is unresolved. V_{OX} is instead measured from the reduction peak at +1088 mV wrt ferrocene reduction at +288 mV.

4.8 – 7,17-Bis(4-hexylphenyl)dinaphtho[1,2,-*b*:1',2'-*k*]chrysene (28)

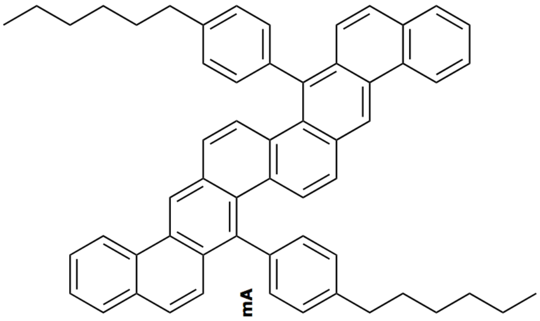

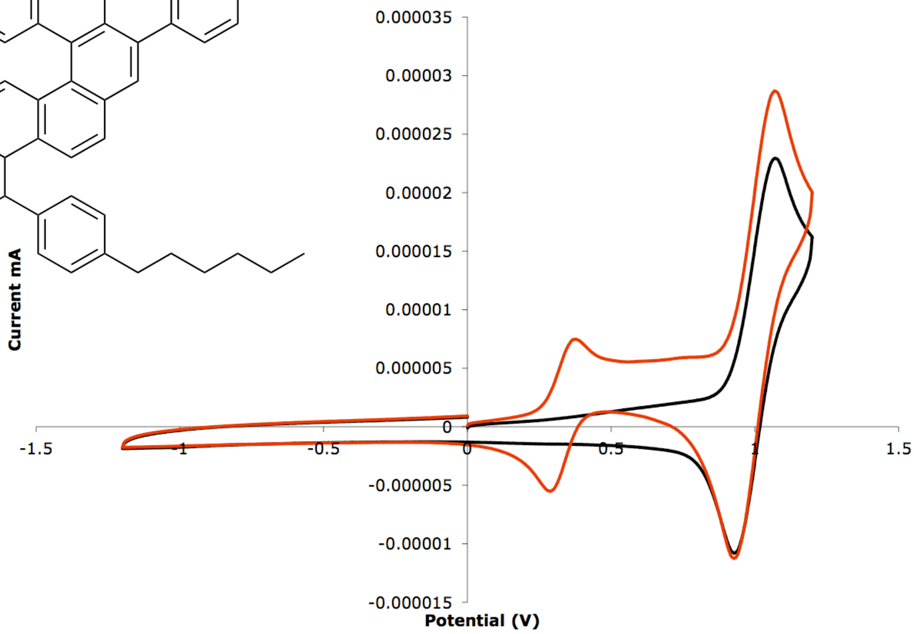

5.1 – ^{1}H and ^{13}C NMR Spectra for Key Compounds

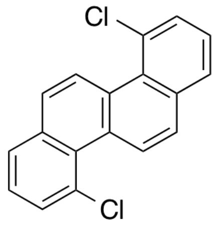

4,10-Dichlorochrysene **5**

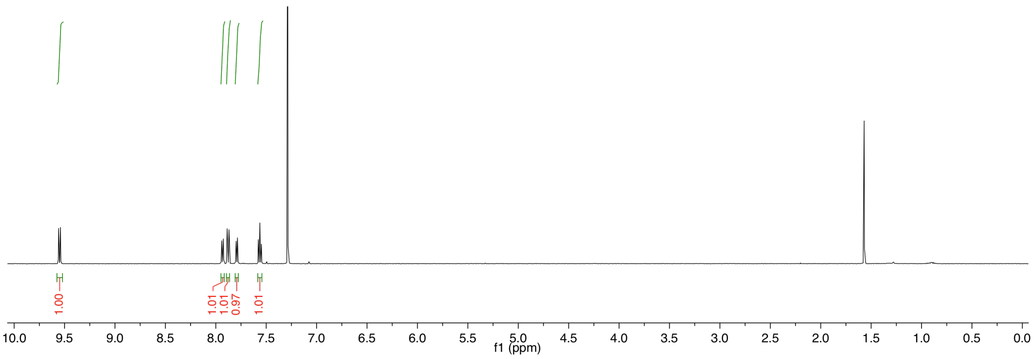

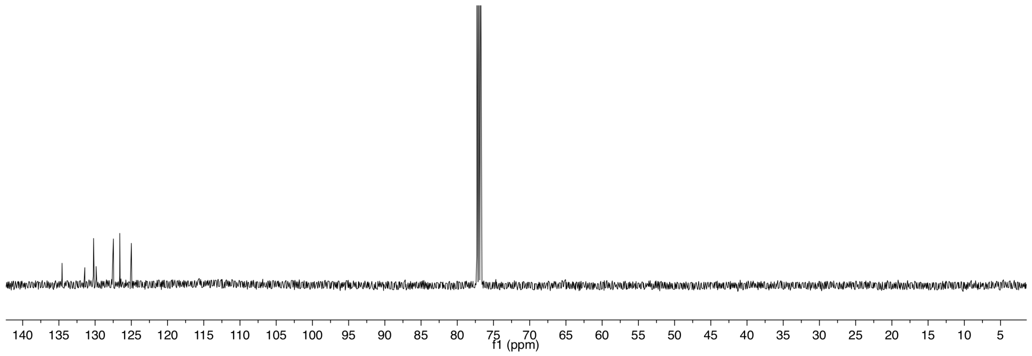

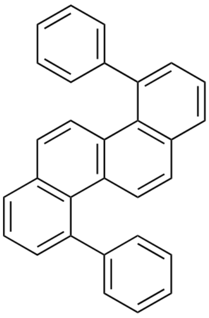

4,10-Diphenylchrysene **6**

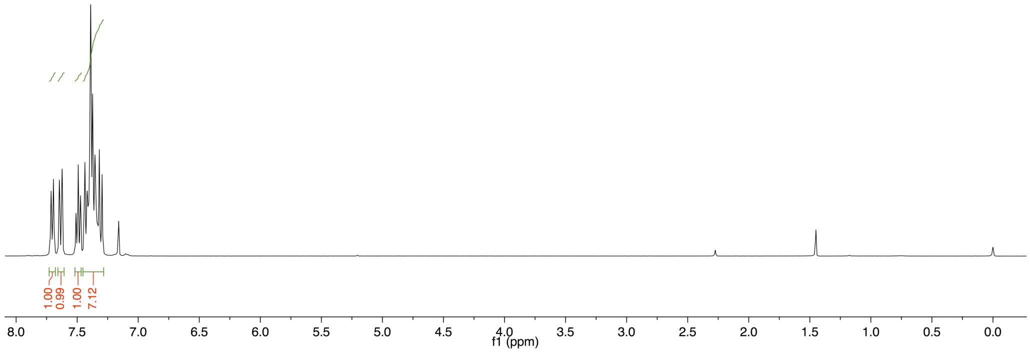

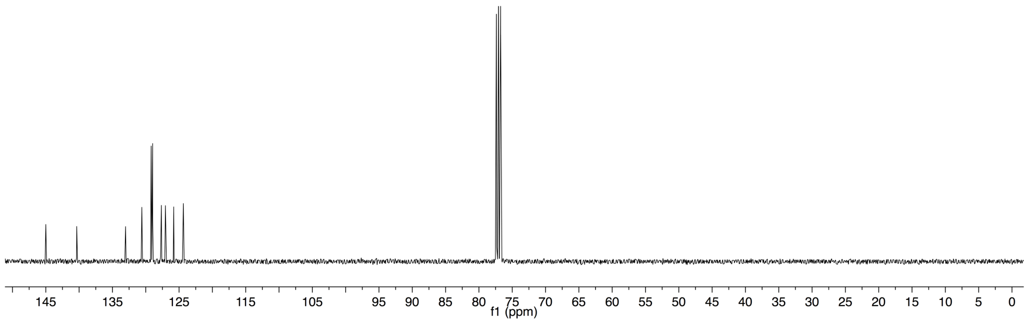

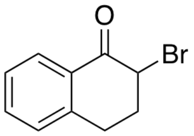

2-Bromo-1-tetralone **14**

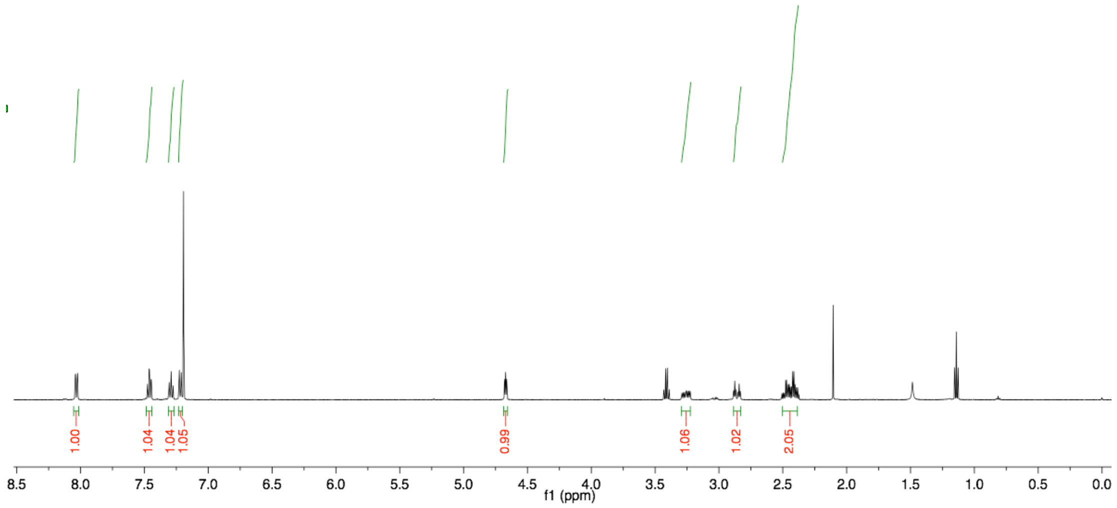

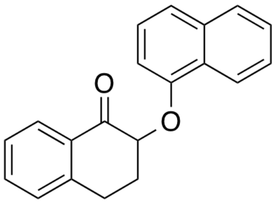

2-(Naphthalene-1′-yloxy)-1-tetralone **15**

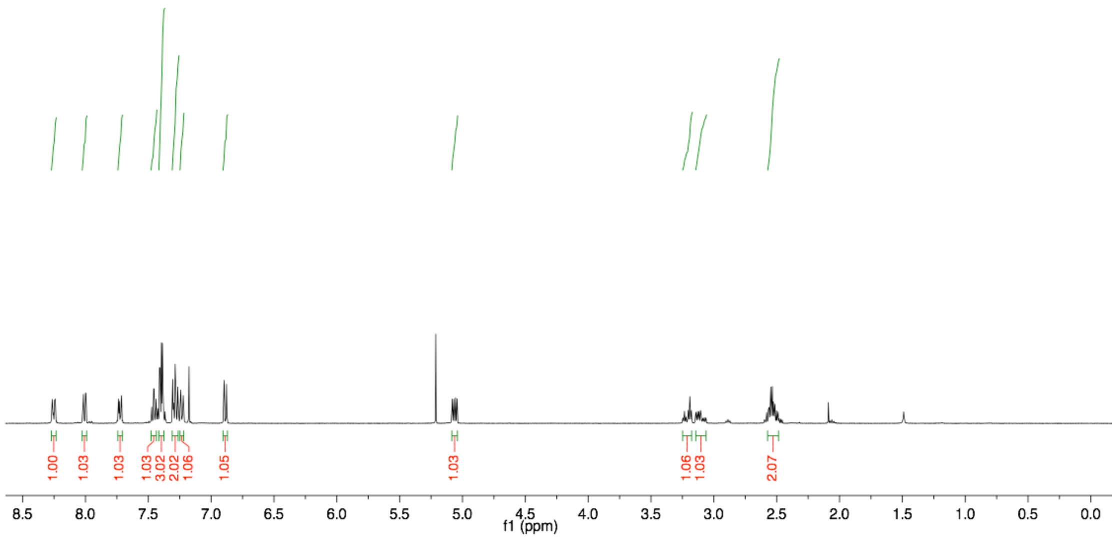

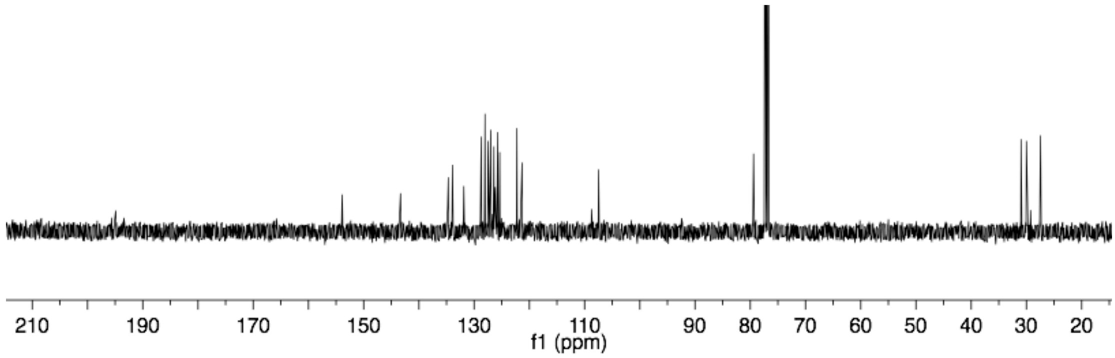

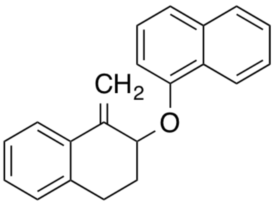

2′-(Naphthalene-1′-yloxy)-1-methylene-2,3,4-trihydronaphthalene **16**

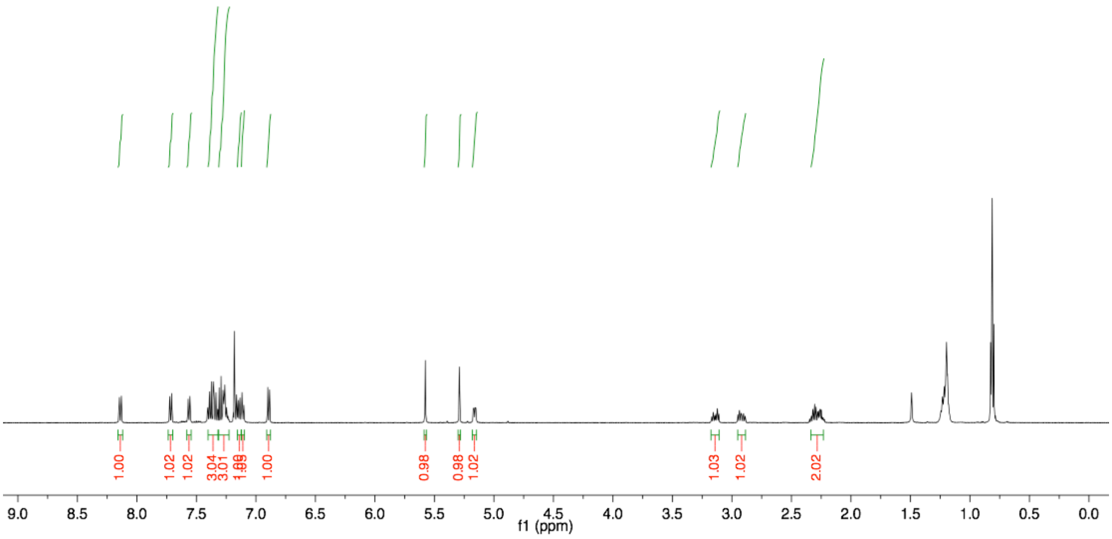

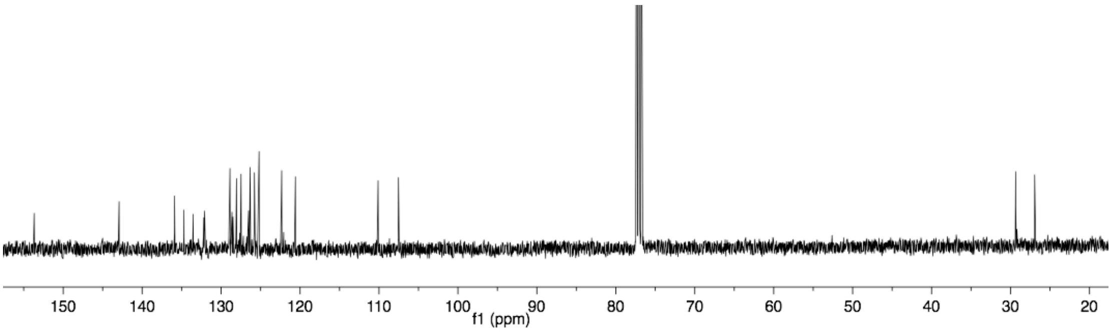

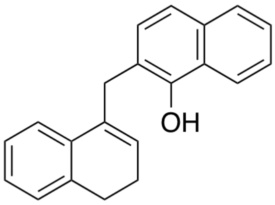

2′-((3,4-Dihydronaphthalen-1-yl)methyl)naphthalen-1′-ol **17**

Phenol 17 undergoes rapid oxidation to the quinone detailed below, precluding isolation and characterisation of the pure material. NMR spectra are therefore of the crude product.

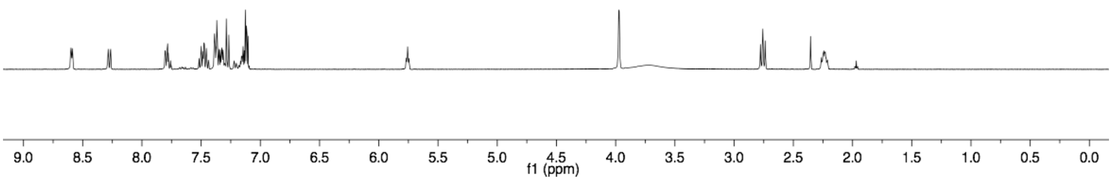

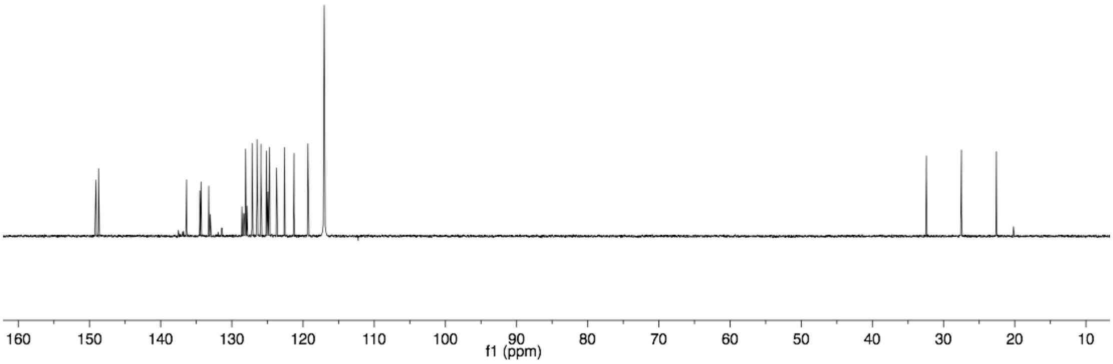

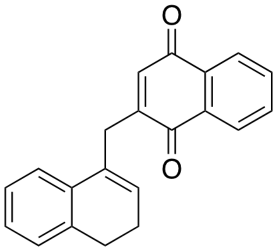

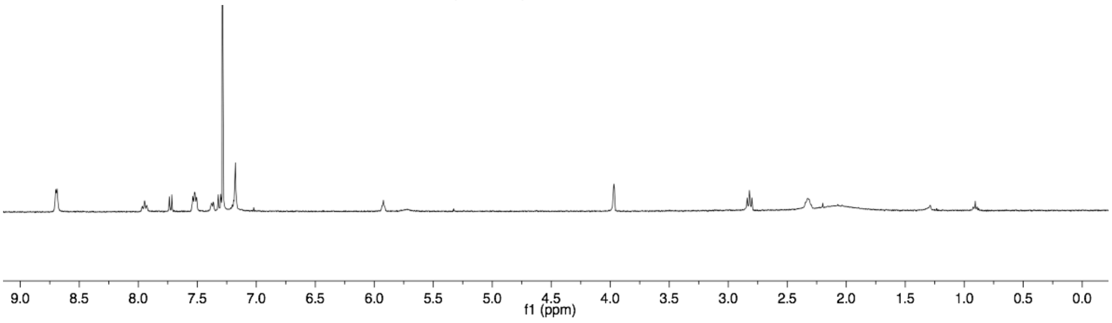

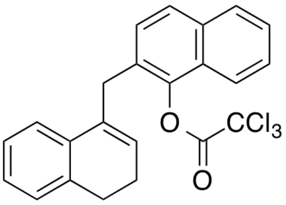

2′-((3,4-Dihydronaphthalen-1-yl)methyl)naphthalen-1′-trichloroacetate **18**

N.B. Trichloroacetate 18 undergoes rapid hydrolysis to 17 and subsequent oxidative decomposition. The following NMR spectra are therefore of the crude product.

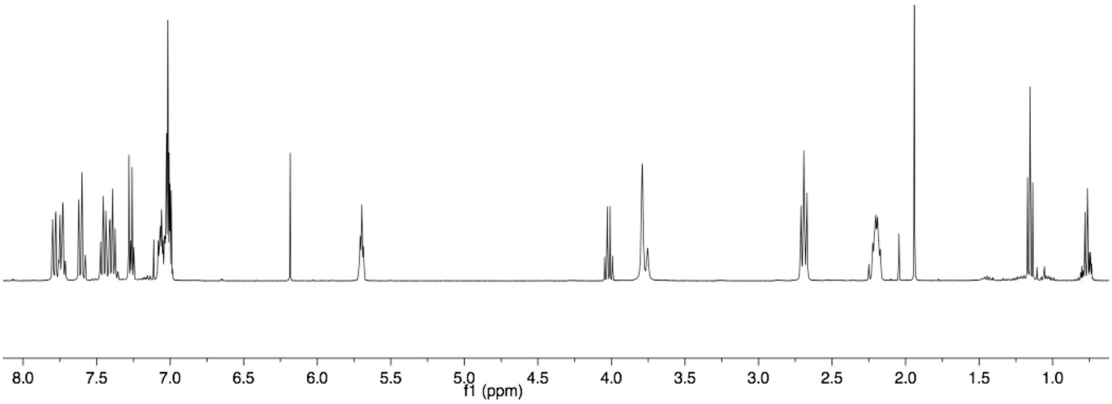

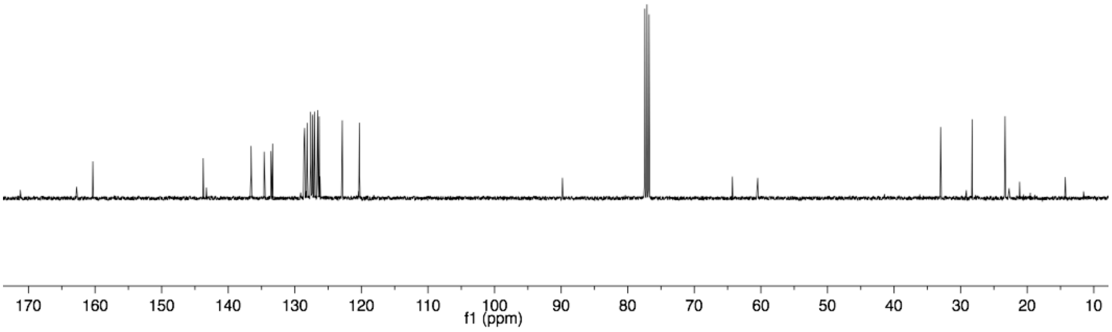

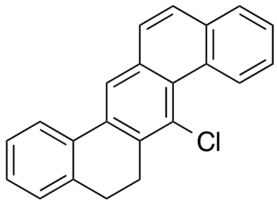

7-Chloro-5,6-dihydrobenzo[k]tetraphene **19**

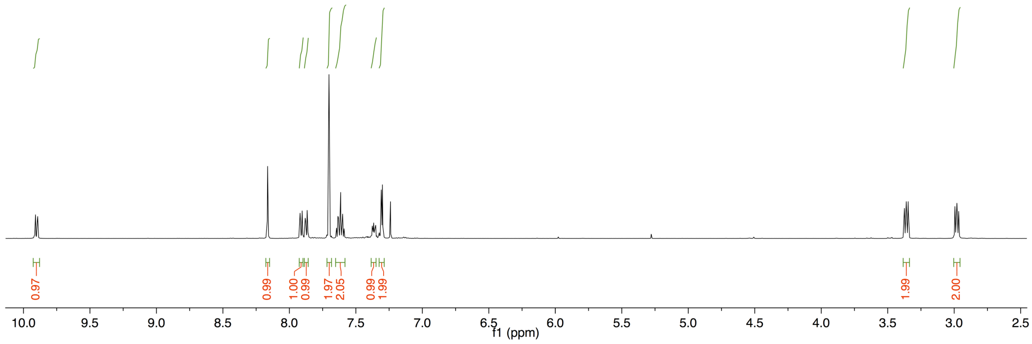

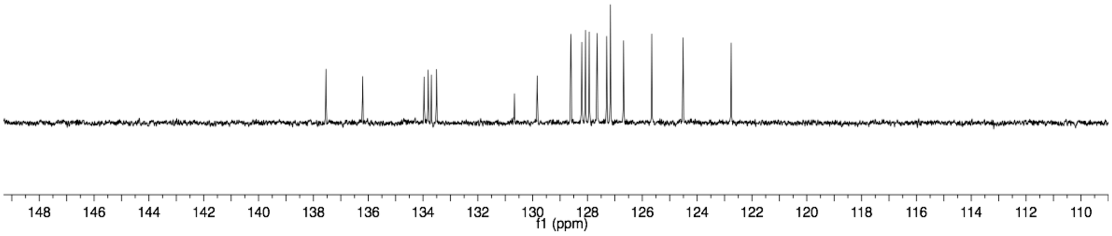

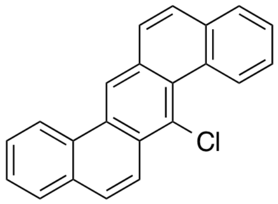

7-Chlorobenzo[k]tetraphene **20**

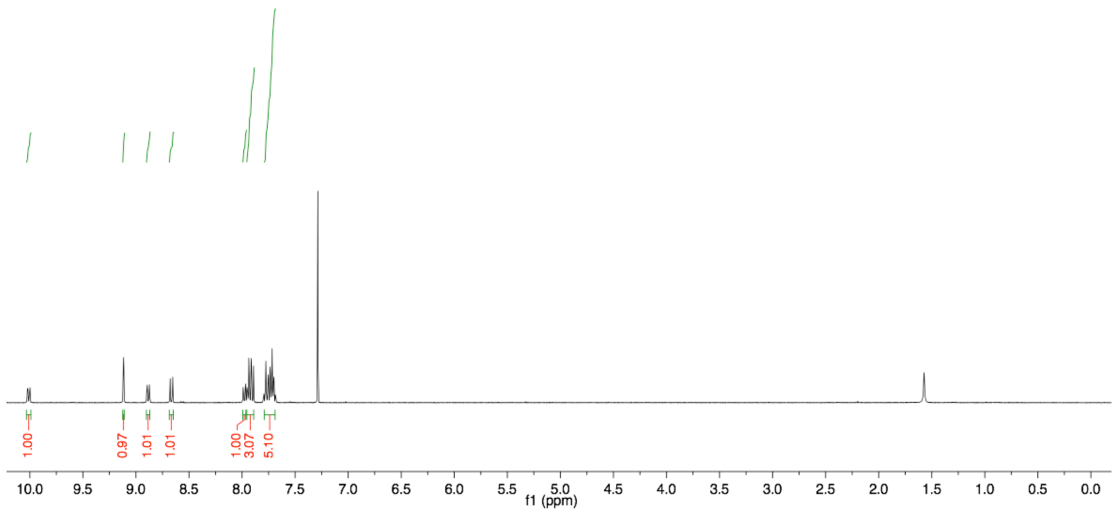

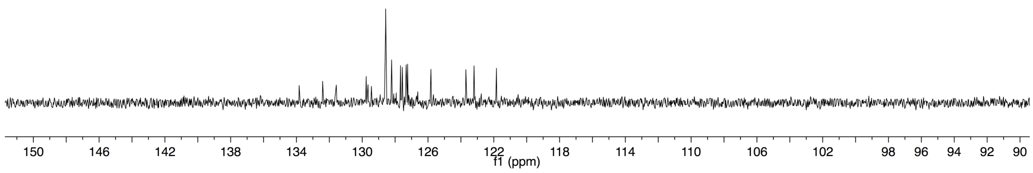

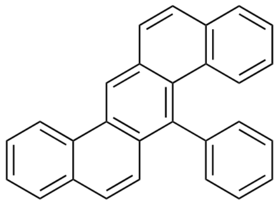

7-Phenylbenzo[k]tetraphene **21**

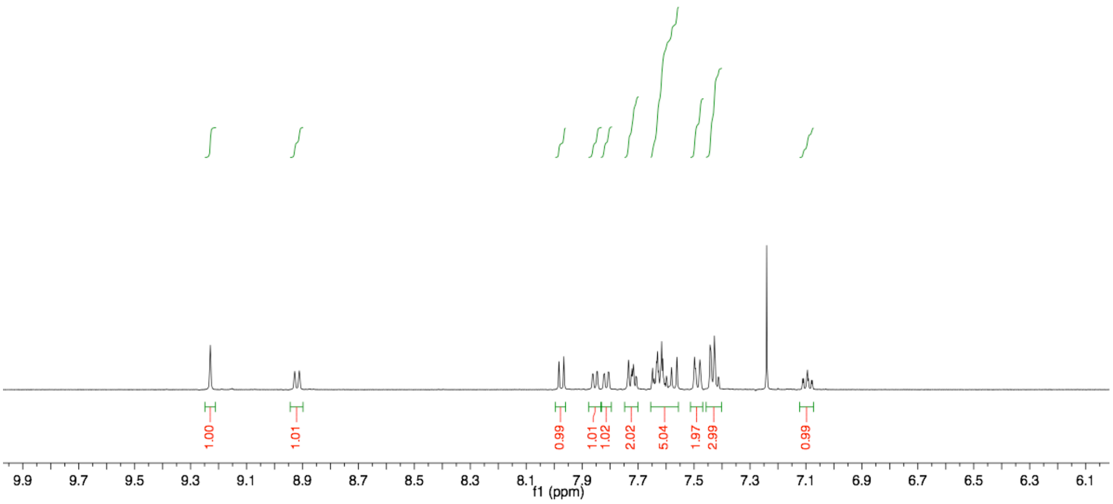

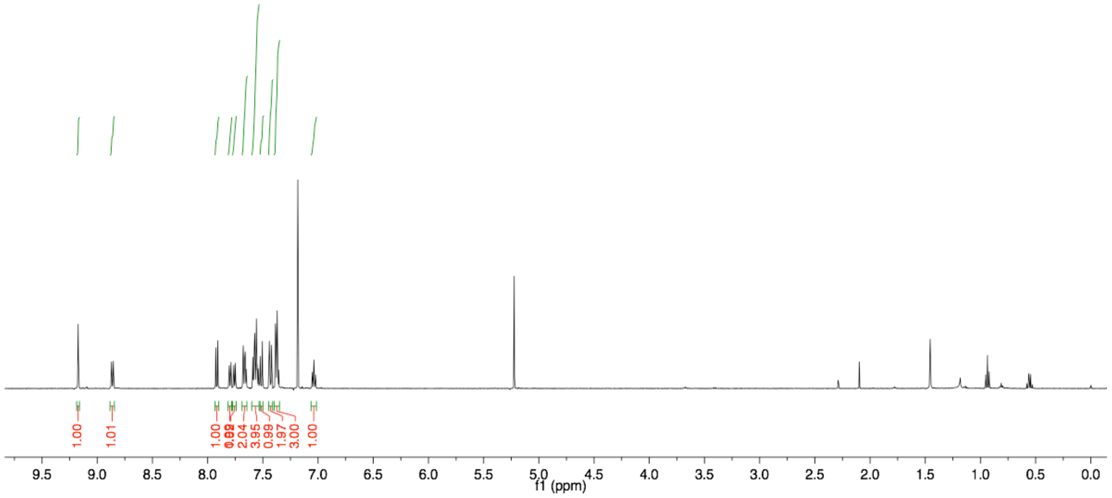

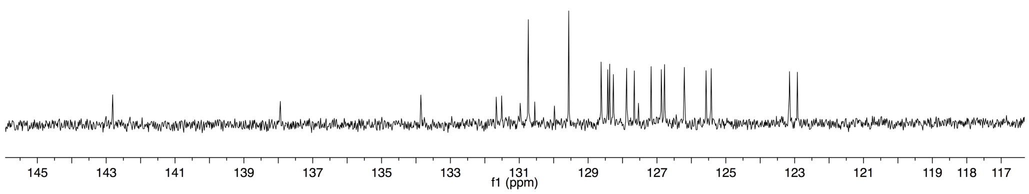

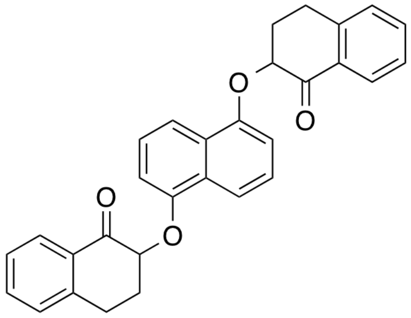

1,5-Bis(2-tetralonoxy)naphthalene **22**

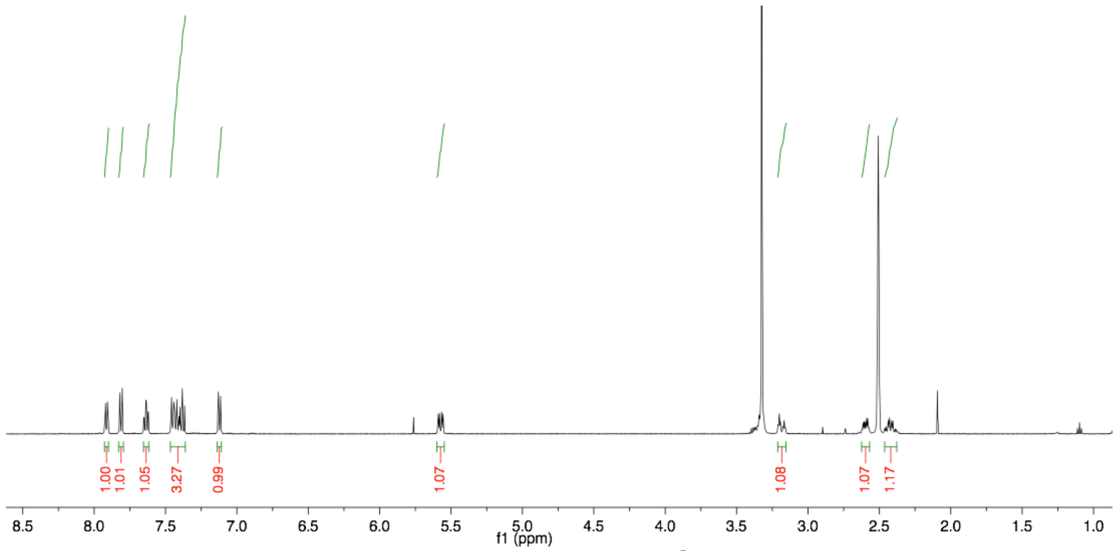

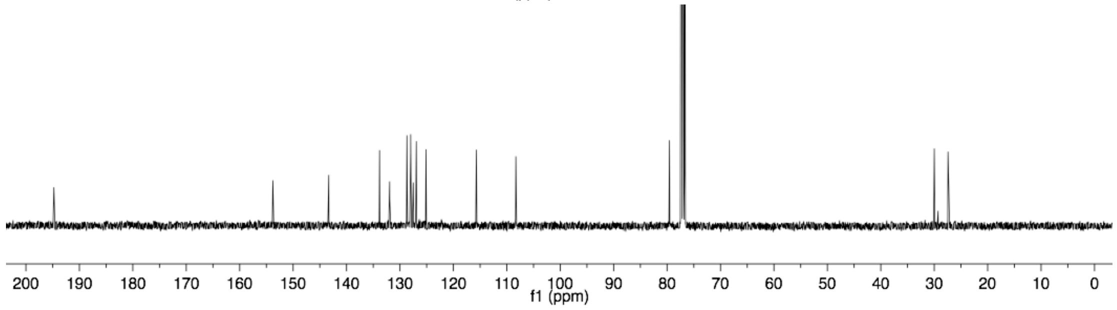

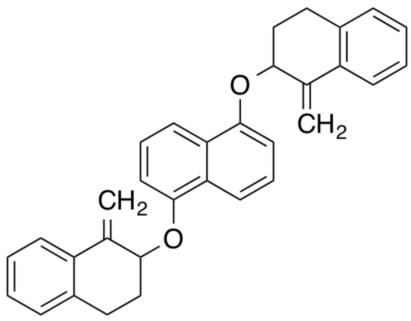

1′,5′-Bis((1-methylene-1,2,3,4-tetrahydronaphthalen-2-yl)oxy)naphthalene **23**

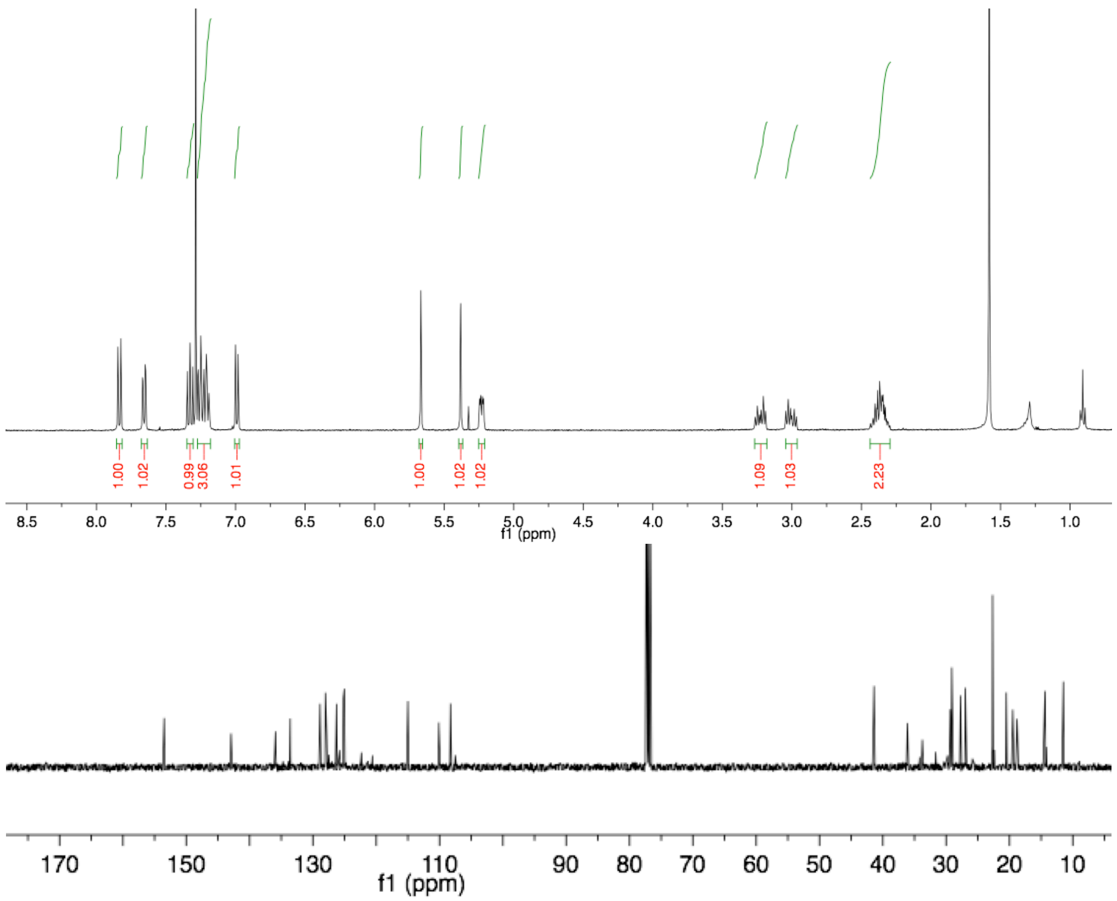

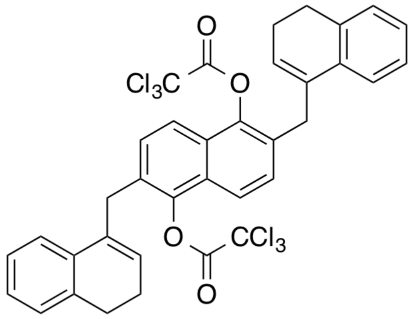

2′,6′-Bis((3,4-dihydronaphthalen-1-yl)methyl)naphthalene-1′,5′-bistrichloroacetate **25**

* indicates DCM and hexane impurities

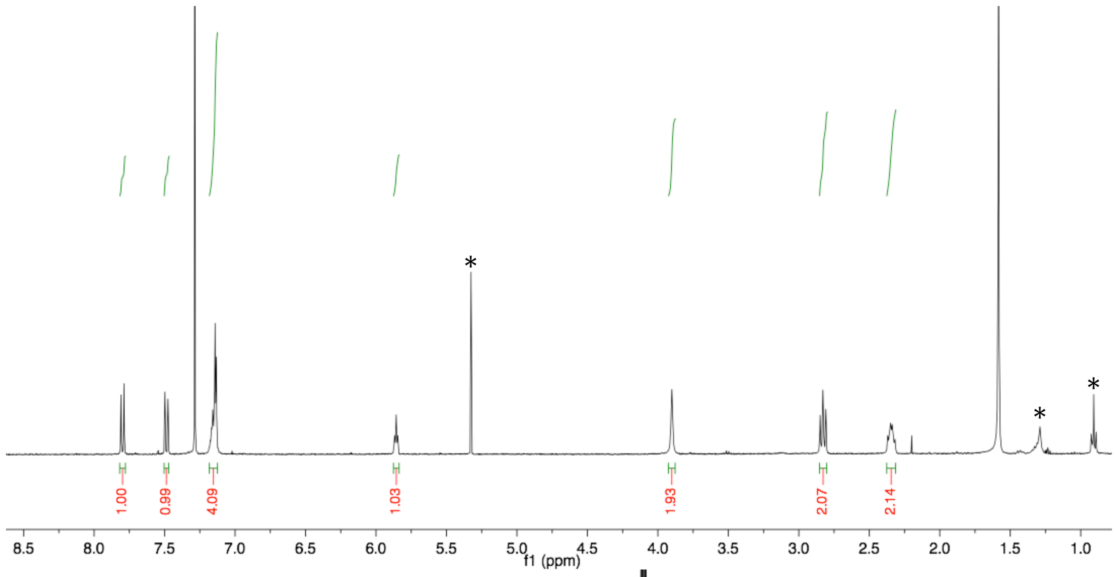

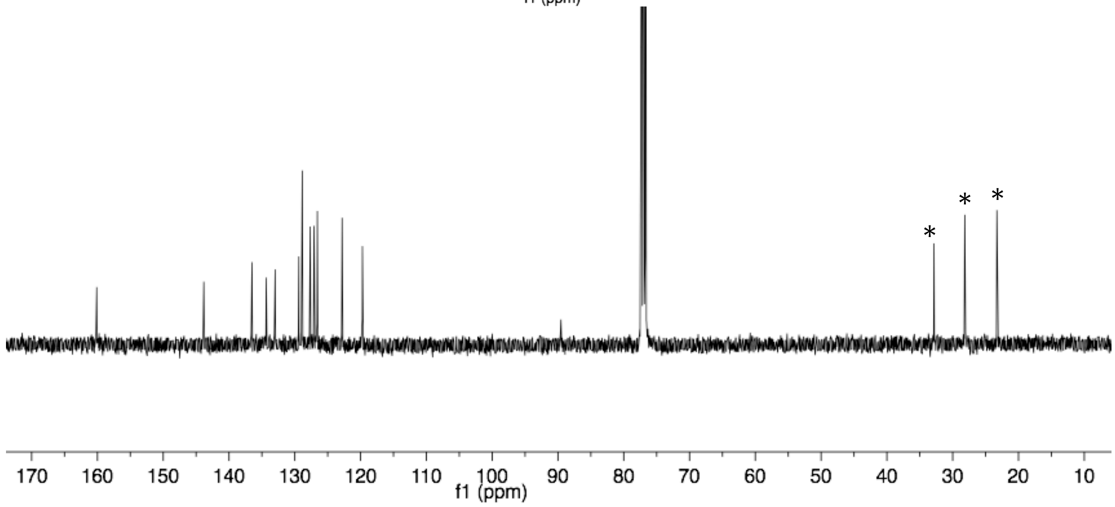

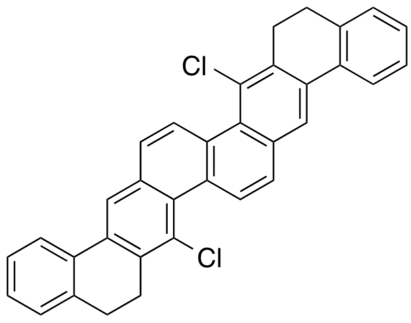

7,17-Dichloro-5,6,15,16-tetrahydrodinaphtho[1,2,-*b*:1',2'-*k*]chrysene **26**

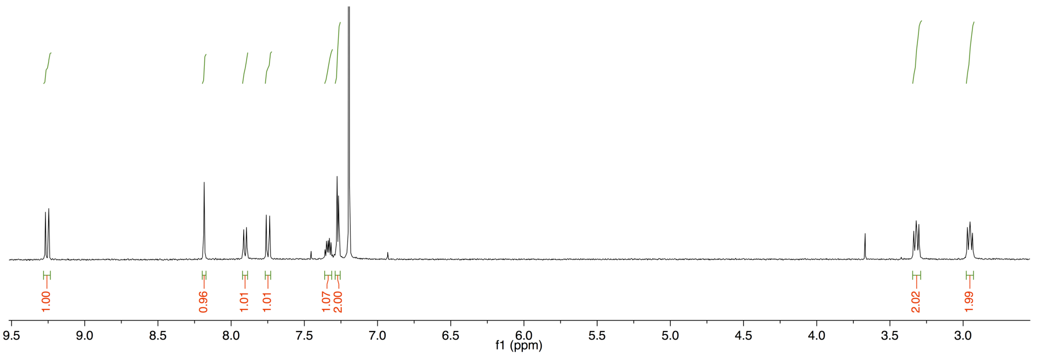

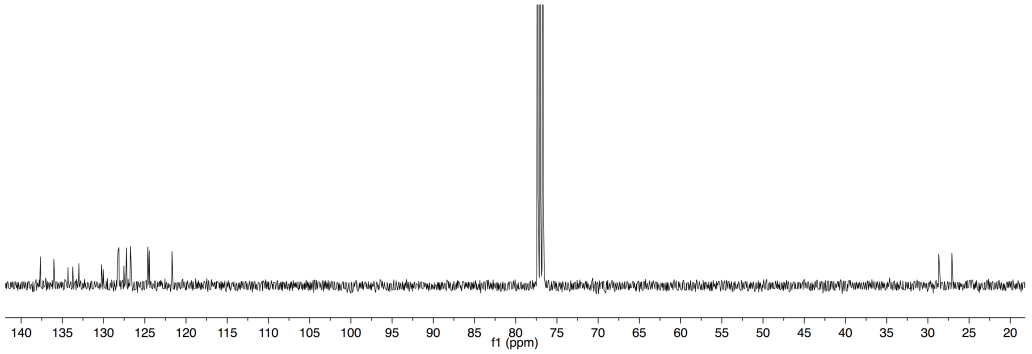

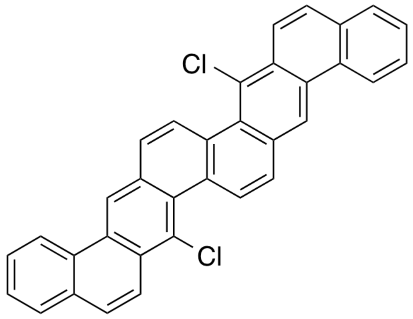

7,17-Dichlorodinaphtho[1,2,-*b*:1',2'-*k*]chrysene **27**

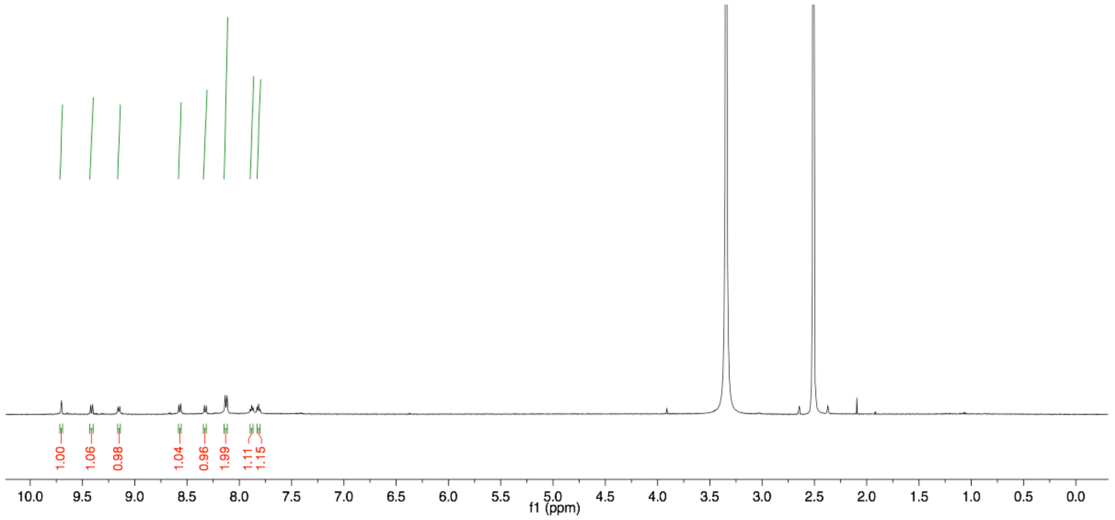

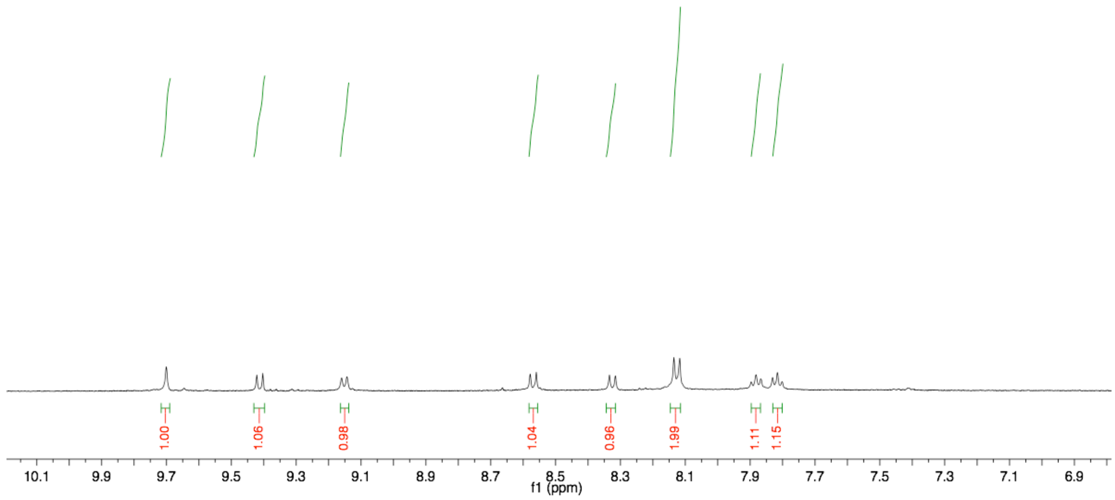

^{13}**C NMR** was unobtainable for this compound due to its poor solubility.

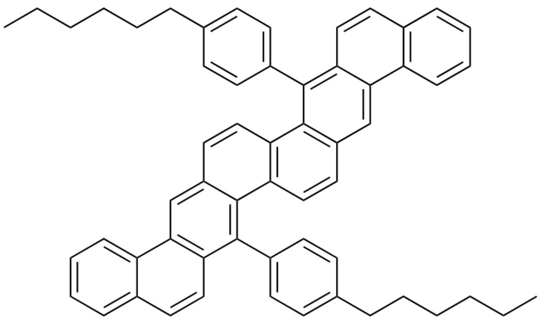

7,17-Bis(4-hexylphenyl)dinaphtho[1,2,-*b*:1',2'-*k*]chrysene **28**

* indicates DCM and hexane impurities

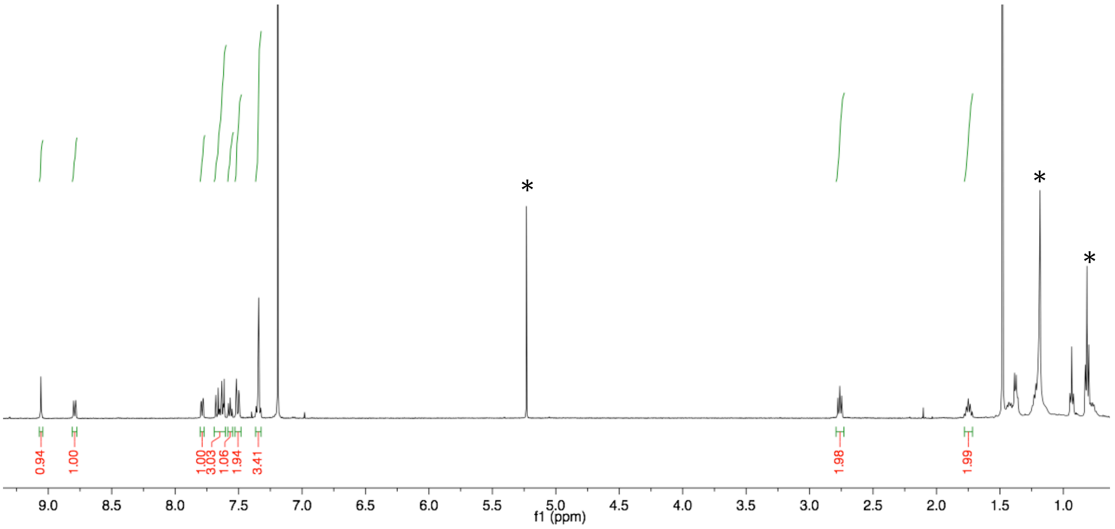

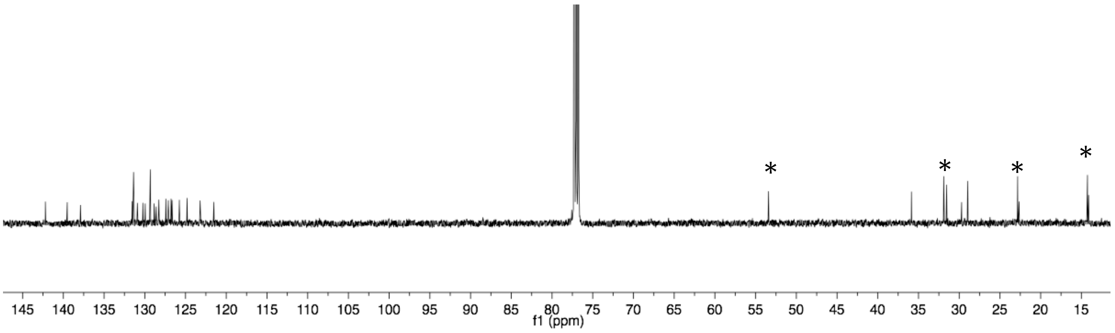

6 – X-Ray Crystal Structure Details

Cif files for the following structures can be obtained free of charge from The Cambridge Crystallographic Data Centre via www.ccdc.cam.ac.uk/data%5Frequest/cif

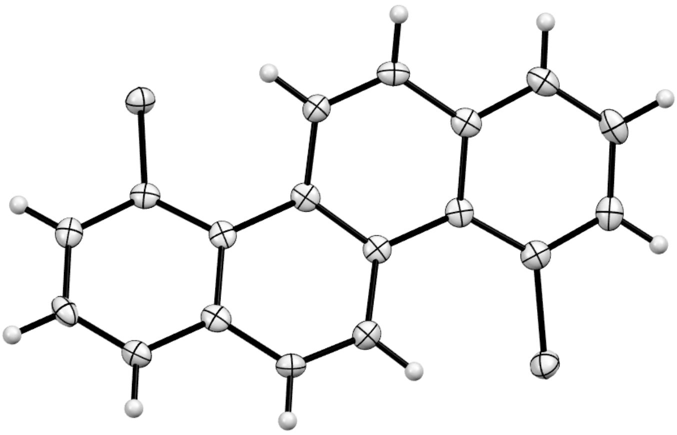

Crystal data for 4,10-dichlorochrysene **5** (CCDC 933450)

UoM Structure code	s3517m	
Empirical formula	C18 H10 Cl2	
Formula weight	297.16	
Temperature	100(2) K	
Wavelength	0.71073 A	
Crystal system, space group	Monoclinic, P21/n	
Unit cell dimensions	a = 16.903(5) A	alpha = 90 deg.
	b = 3.7551(12) A	beta = 109.136(6) deg.
	c = 20.759(6) A	gamma = 90 deg.
Volume	1244.9(7) A^3	
Z, Calculated density	4, 1.586 Mg/m^3	
Absorption coefficient	0.504 mm^-1	
F(000)	608	
Crystal size	0.50 x 0.32 x 0.15 mm	
Theta range for data collection	2.08 to 26.33 deg.	
Limiting indices	-21<=h<=16, -4<=k<=4, -25<=l<=24	
Reflections collected / unique	6480 / 2522 [R(int) = 0.0621]	
Completeness to theta = 26.33	99.4 %	
Absorption correction	None	
Refinement method	Full-matrix least-squares on F^2	
Data / restraints / parameters	2522 / 0 / 181	
Goodness-of-fit on F^2	0.926	
Final R indices [I>2sigma(I)]	R1 = 0.0504, wR2 = 0.1129	
R indices (all data)	R1 = 0.0720, wR2 = 0.1220	
Largest diff. peak and hole	0.620 and -0.344 e.A^-3	

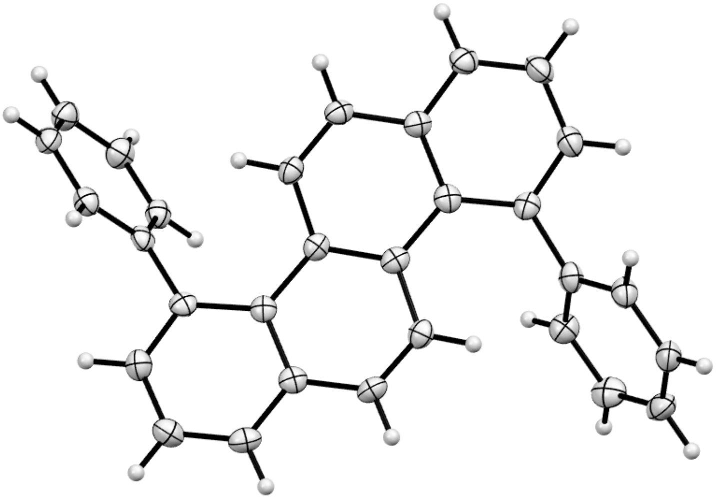

Crystal data for 4,10-diphenylchrysene **6** (CCDC 933452)

UoM Structure code	s3524m	
Empirical formula	C30 H20	
Formula weight	380.46	
Temperature	100(2) K	
Wavelength	0.71073 A	
Crystal system, space group	Triclinic, P-1	
Unit cell dimensions	a = 10.2889(13) A	alpha = 70.719(2) deg.
	b = 11.8850(15) A	beta = 75.589(2) deg.
	c = 17.653(2) A	gamma = 82.236(2) deg.
Volume	1970.2(4) A^3	
Z, Calculated density	4, 1.283 Mg/m^3	
Absorption coefficient	0.073 mm^-1	
F(000)	800	
Crystal size	0.30 x 0.25 x 0.20 mm	
Theta range for data collection	2.05 to 26.40 deg.	
Limiting indices	-12<=h<=12, -14<=k<=14, -22<=l<=22	
Reflections collected / unique	15686 / 7916 [R(int) = 0.0534]	
Completeness to theta = 25.00	98.9 %	
Absorption correction	None	
Max. and min. transmission	0.9856 and 0.9786	
Refinement method	Full-matrix least-squares on F^2	
Data / restraints / parameters	7916 / 0 / 541	
Goodness-of-fit on F^2	0.888	
Final R indices [I>2sigma(I)]	R1 = 0.0580, wR2 = 0.1043	
R indices (all data)	R1 = 0.1049, wR2 = 0.1214	
Largest diff. peak and hole	0.228 and -0.199 e.A^-3	

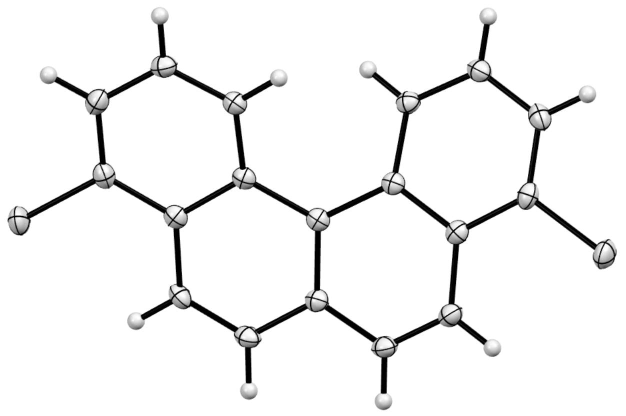

Crystal data for 4,9-dichlorobenzo[c]phenanthrene **11** (CCDC 1040409)

UoM Structure code	s3998ma	
Empirical formula	C18 H10 Cl2	
Formula weight	297.16	
Temperature	150(2) K	
Wavelength	1.54178 A	
Crystal system, space group	Monoclinic, P2(1)/c	
Unit cell dimensions	a = 3.79030(10) A	alpha = 90 deg.
	b = 22.3184(4) A	beta = 93.2450 deg.
	c = 14.9033(2) A	gamma = 90 deg.
Volume	1258.7(4) A^3	
Z, Calculated density	4, 1.568 Mg/m^3	
Absorption coefficient	4.482 mm^-1	
F(000)	608	
Crystal size	0.26 x 0.12 x 0.07 mm	
Theta range for data collection	3.57 to 72.19 deg	
Limiting indices	-4<=h<=3, -27<=k<=26, -18<=l<=17	
Reflections collected / unique	7001 / 2427 [R(int) = 0.0203]	
Completeness to theta = 67.00	97.6 %	
Absorption correction	Semiempirical from equivalents	
Max. and min. transmission	0.7444 and 0.640978	
Refinement method	Full-matrix least-squares on F^2	
Data / restraints / parameters	2427 / 0 / 181	
Goodness-of-fit on F^2	1.070	
Final R indices [I>2sigma(I)]	R1 = 0.0270, wR2 = 0.0726	
R indices (all data)	R1 = 0.0283, wR2 = 0.0734	
Largest diff. peak and hole	0.249 and -0.241 e.A^-3	

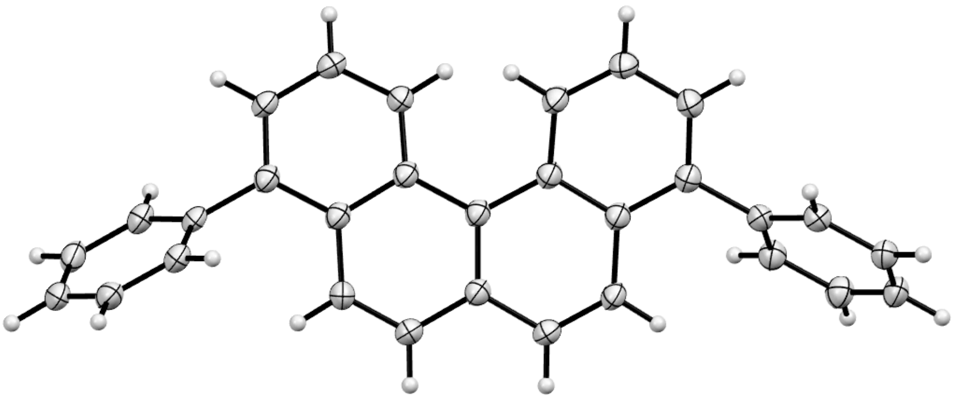

Crystal data for 4,9-diphenylbenzo[c]phenanthrene **12** (CCDC 1040408)

UoM Structure code	s4126na	
Empirical formula	C30 H20	
Formula weight	380.46	
Temperature	100(2) K	
Wavelength	1.5418 A	
Crystal system, space group	Triclinic, P -1	
Unit cell dimensions	a = 9.4832(5) A	alpha = 76.453(4) deg.
	b = 9.6223(5) A	beta = 73.874(5) deg.
	c = 11.9463(5) A	gamma = 70.737(5) deg.
Volume	976.28(9) A^3	
Z, Calculated density	2, 1.294 Mg/m^3	
Absorption coefficient	0.555 mm^-1	
F(000)	400	
Crystal size	0.29 x 0.23 x 0.14 mm	
Theta range for data collection	4.93 to 74.00 deg	
Limiting indices	-11<=h<=11, -8<=k<=11, -14<=l<=14	
Reflections collected / unique	9971 / 3734 [R(int) = 0.0327]	
Completeness to theta = 67.00	97.0 %	
Absorption correction	Semiempirical from equivalents	
Max. and min. transmission	1.00000 and 0.46686	
Refinement method	Full-matrix least-squares on F^2	
Data / restraints / parameters	3734 / 0 / 271	
Goodness-of-fit on F^2	1.056	
Final R indices [I>2sigma(I)]	R1 = 0.0531, wR2 = 0.1501	
R indices (all data)	R1 = 0.0568, wR2 = 0.1562	
Largest diff. peak and hole	0.272 and -0.266 e.A^-3	

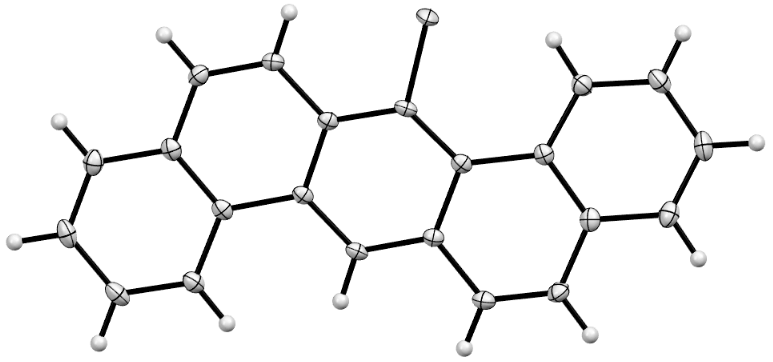

Crystal data for 7-chlorobenzo[k]tetraphene **20** (CCDC 1040540)

UoM Structure code	s3774ma	
Empirical formula	C22 H13 Cl	
Formula weight	312.77	
Temperature	100(2) K	
Wavelength	1.54178 Å	
Crystal system	Monoclinic	
Space group	P2(1)/c	
Unit cell dimensions\	a = 7.7610 (3)	α = 90°.
	b = 11.2321 (5)	β = 90.228 (2)°.
	c = 16.5854 (8)	γ = 90°.
Volume	1445.78 (11) $Å^3$	
Density (calculated)	1.437 Mg/m^3	
Absorption coefficient	2.276 mm^{-1}	
F(000)	648	
Crystal size	0.22 x 0.18 x 0.04 mm^3	
Theta range for data collection	4.75 to 72.19°	
Index ranges	-9<=h<=9, -13=k<=13, -19<=l<=18	
Reflections collected	9080	
Independent reflections	2783 [R(int) = 0.0307]	
Completeness to theta = 67.00°	98.4 %	
Max. and min. transmission	0.9145 and 0.642796	
Refinement method	Full-matrix least-squares on F^2	
Data / restraints / parameters	2783 / 0 / 208	
Goodness-of-fit on F^2	1.067	
Final R indices [I>2sigma(I)]	R1 = 0.0374, wR2 = 0.1002	
R indices (all data)	R1 = 0.0387, wR2 = 0.1016	
Largest diff. peak and hole	0.410 and -0.321 e.$Å^{-3}$	

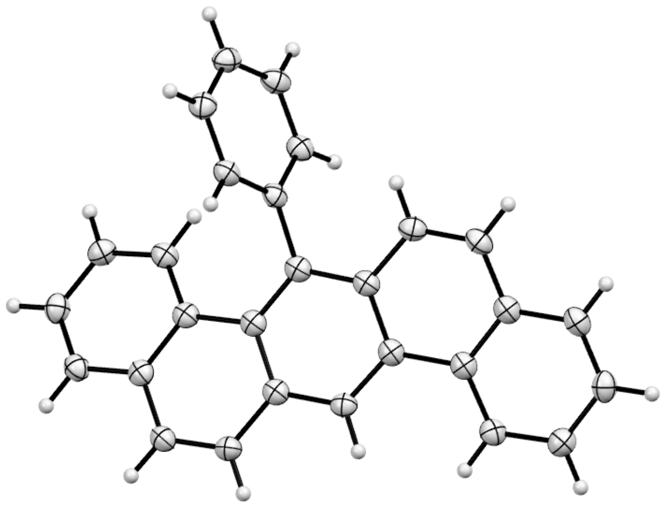

Crystal data for 7-phenylbenzo[k]tetraphene **21** (CDC 1040410)

UoM Structure code	s3969ma	
Empirical formula	C28 H18	
Formula weight	354.42	
Temperature	100(2) K	
Wavelength	1.54178	
Crystal system	Orthorhombic	
Space group	Pbca	
Unit cell dimensions\	a = 10.4773(3)	α = 90°.
	b = 17.8272(5)	β = 90°.
	c = 18.9595(6)	γ = 90°.
Volume	3541.27(18) $Å^3$	
Density (calculated)	1.330 Mg/m^3	
Absorption coefficient	0.571 mm^{-1}	
F(000)	1488	
Crystal size	0.29 x 0.27 x 0.10 mm^3	
Theta range for data collection	4.96 to 72.54°	
Index ranges	-12<=h<=12, -22<=k<=20, -22<=l<=18	
Reflections collected	17139	
Independent reflections	3452 [R(int) = 0.0591]	
Completeness to theta = 67.00°	99.2 %	
Max. and min. transmission	0.9451 and 0.654899	
Refinement method	Full-matrix least-squares on F^2	
Data / restraints / parameters	3452 / 0 / 254	
Goodness-of-fit on F^2	1.055	
Final R indices [I>2sigma(I)]	R1 = 0.0475, wR2 = 0.1290	
R indices (all data)	R1 = 0.0519, wR2 = 0.1333	
Extinction coefficient	0.0016(3)	
Largest diff. peak and hole	0.274 and -0.201 e.$Å^{-3}$	

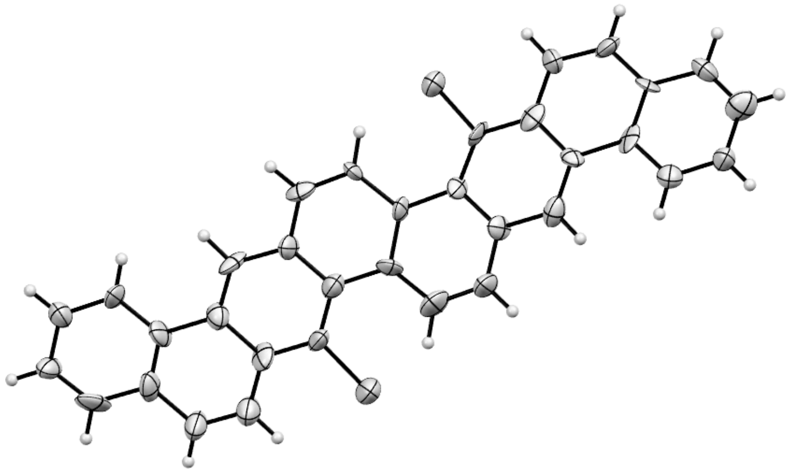

Crystal data for 7,17-dichlorodinaphtho[1,2,-b:1',2'-k]chrysene **27** (CCDC 1040773)

UoM Structure code	xrgp102	
Empirical formula	C34 H18 Cl2	
Formula weight	497.38	
Temperature	100(2) K	
Wavelength	0.71073 Å	
Crystal system	Monoclinic	
Space group	P 2_1/c	
Unit cell dimensions	a = 23.152(6) Å	α = 90°.
	b = 3.763(2) Å	β = 100.81(3)°.
	c = 24.879(9) Å	γ = 90°.
Volume	2129.3(16) $Å^3$	
Z	4	
Density (calculated)	1.552 Mg/m^3	
Absorption coefficient	0.330 mm^{-1}	
F(000)	1024	
Crystal size	0.15 x 0.06 x 0.02 mm^3	
Theta range for data collection	2.96 to 25.50°.	
Index ranges	-28<=h<=19, -4<=k<=4, -26<=l<=30	
Reflections collected	8949	
Independent reflections	3945 [R(int) = 0.2823]	
Completeness to theta = 25.50°	98.8 %	
Max. and min. transmission	0.9934 and 0.9521	
Refinement method	Full-matrix least-squares on F^2	
Data / restraints / parameters	3945 / 204 / 325	
Goodness-of-fit on F^2	0.967	
Final R indices [I>2sigma(I)]	R1 = 0.1166, wR2 = 0.1464	
R indices (all data)	R1 = 0.3636, wR2 = 0.2370	
Largest diff. peak and hole	0.397 and -0.346 e.$Å^{-3}$	

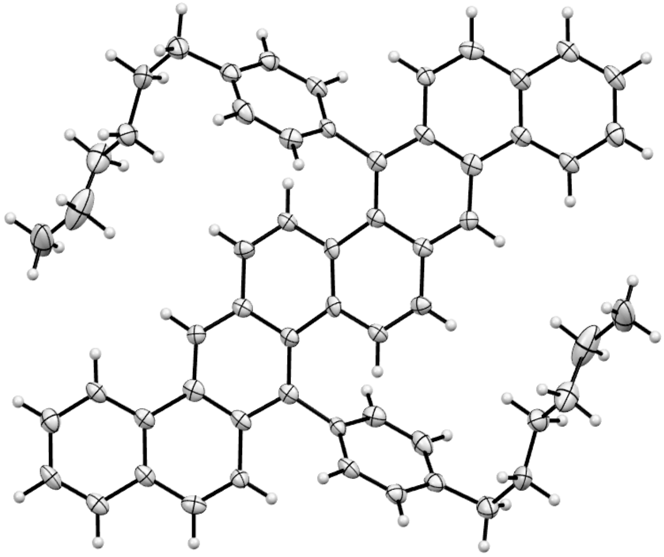

Crystal data for 7,17-dichlorodinaphtho[1,2,-b:1',2'-k]chrysene **28** (CCDC 1040411)

UoM Structure code	s4141na	
Empirical formula	C58 H52	
Formula weight	749.00	
Temperature	100(2) K	
Wavelength	1.5418 Å	
Crystal system	Tricyclic	
Space group	P -1	
Unit cell dimensions	a = 6.0573(4) Å	α = 108.089(7)°.
	b = 17.1347(13) Å	β = 90.153(6)°.
	c = 20.6747(15) Å	γ = 99.154(6)°.
Volume	2010.7(3) $Å^3$	
Z	2	
Density (calculated)	1.237 Mg/m^3	
Absorption coefficient	0.522 mm^{-1}	
F(000)	80	
Crystal size	0.22 x 0.04 x 0.01 mm^3	
Theta range for data collection	2.75 to 74.21°.	
Index ranges	-7<=h<=7, -21<=k<=20, -22<=l<=25	
Reflections collected	21423	
Independent reflections	7694 [R(int) = 0.2174]	
Completeness to theta = 25.50°	97.0 %	
Max. and min. transmission	1.00000 and 0.77843	
Refinement method	Full-matrix least-squares on F^2	
Data / restraints / parameters	7694 / 6 / 564	
Goodness-of-fit on F^2	1.015	
Final R indices [I>2sigma(I)]	R1 = 0.0931, wR2 = 0.1804	
R indices (all data)	R1 = 0.2268, wR2 = 0.2588	
Largest diff. peak and hole	0.330 and -0.354 e.$Å^{-3}$	

7 - Field Effect Transistors

Field effect transistors were created for compound **28**. The thin films were characterised by X-ray diffraction and atomic force microscopy and transistors were created and analysed using conventional thin film transistor techniques.

7.1 - Thin Film Deposition

All materials and devices were prepared on heavily doped silicon (n++) substrates with 300 nm of thermally grown silicon dioxide. The substrates were cleaned by washing and sonication in acetone, propan-2-ol and methanol followed by UV-Ozone treatment. The surfaces of the substrates were then treated with octadecyltrichlorosilane (OTS) monolayers to reduce the surface energy, passivate traps and improve thin film growth. OTS treatment was done by spin coating of an OTS solution from chloroform as reported elsewhere.[1] OTS treated substrates were all washed with organic solvents and dried with nitrogen before being transferred to the vacuum chamber for thin film deposition.

Evaporation of the organic semiconductor was performed in a modified Edwards Auto306 vacuum evaporator with a base pressure of 7×10^{-6} mbar. Nominally 30 nm was deposited onto substrates held at 40 °C at a rate of 0.5 As^{-1}

To create thin film transistors the as prepared substrates were transferred to a separate Edwards Auto500 vacuum evaporator (base pressure 1×10^{-7} mbar) for gold evaporation. Nominally 50 nm of gold was deposited on top of the organic layers at a rate of 1 As^{-1} through a shadow mask.

7.2 - Thin Film Characterisation

The thin films were characterised using a Park XE100 atomic force microscope in tapping mode and a Bruker D8 discover X-ray diffractometer for out-of-plane X-ray diffraction. The compound creates small crystals approximately 0.25 μm^2 large with a thin film roughness of 2.1 nm. The out-of-plane x-ray diffraction allows us to calculate the relative tilt of the molecule to the substrate surface by comparing the 001 reflection with the calculated molecular length from single

crystal x-ray diffraction. Using Bragg's law the d-spacing is calculated to be 1.67 nm, using the calculated molecular length of 2.07 nm suggests that the molecule is tilted toward the surface normal at an angle of 36 °.

7.3 - Transistor Characterisation

A series of 10 transistors were created via the evaporation of gold electrodes in a top contact bottom gate configuration. The devices were then tested in the saturation regime using the standard equation.

$$I_D = C_i \frac{W}{2L} \mu_{sat} (V_G - V_T)^2$$

Where, I_D is the source-drain current, C_i is the capacitance, W the channel width, L the channel length, μ_{sat} is the saturation mobility, V_G is the swept gate voltage and V_T is the threshold voltage. The transistors had a channel width of 2000 μm and a channel length of 60 μm, the 300 nm silicon dioxide with OTS monolayer was calculated to have a capacitance of approximately 11.4 nFcm^{-2}. The devices showed good transistor behaviour with an average saturation mobility of 0.03 ± 0.01 cm^{2}V^{-1}s^{-1}, a threshold of -9 ± 1 V, on/off ratio of 4 x 10^6 and a subthreshold swing of 600 mVdec^{-1}.
